# YAP–TEAD1 control of cytoskeleton dynamics and intracellular tension guides human pluripotent stem cell mesoderm specification

**DOI:** 10.1038/s41418-020-00643-5

**Published:** 2020-10-28

**Authors:** Stefania Pagliari, Vladimir Vinarsky, Fabiana Martino, Ana Rubina Perestrelo, Jorge Oliver De La Cruz, Guido Caluori, Jan Vrbsky, Pamela Mozetic, Antonio Pompeiano, Andrea Zancla, Sri Ganji Ranjani, Petr Skladal, Dan Kytyr, Zbyněk Zdráhal, Gabriele Grassi, Maurilio Sampaolesi, Alberto Rainer, Giancarlo Forte

**Affiliations:** 1 International Clinical Research Center (ICRC) of St Anne’s University Hospital, CZ-65691 Brno, Czech Republic; 2 Competence Center for Mechanobiology in Regenerative Medicine, INTERREG ATCZ133, CZ-62500 Brno, Czech Republic; 3 Faculty of Medicine, Department of Biology, Masaryk University, CZ-62500 Brno, Czech Republic; 4 Central European Institute of Technology, Masaryk University, CZ-62500 Brno, Czech Republic; 5 Università Campus Bio-Medico di Roma, Rome, Italy; 6 Czech Academy of Sciences, Institute of Theoretical and Applied Mechanics, 190 00 Prague 9, Czech Republic; 7 Department of Life Sciences, Cattinara University Hospital, Trieste University, I-34149 Trieste, Italy; 8 Department of Development and Regeneration, KU Leuven, 3000 Leuven, Belgium; 9 Institute of Nanotechnology (NANOTEC), National Research Council, c/o Campus EcoTekne, via Monteroni, 73100 Lecce, Italy; 10 Department of Biomaterials Science, Institute of Dentistry, University of Turku, FI-20014 Turku, Finland

**Keywords:** Extracellular matrix, Molecular biology

## Abstract

The tight regulation of cytoskeleton dynamics is required for a number of cellular processes, including migration, division and differentiation. YAP–TEAD respond to cell–cell interaction and to substrate mechanics and, among their downstream effects, prompt focal adhesion (FA) gene transcription, thus contributing to FA-cytoskeleton stability. This activity is key to the definition of adult cell mechanical properties and function. Its regulation and role in pluripotent stem cells are poorly understood. Human PSCs display a sustained basal YAP-driven transcriptional activity despite they grow in very dense colonies, indicating these cells are insensitive to contact inhibition. PSC inability to perceive cell–cell interactions can be restored by tampering with Tankyrase enzyme, thus favouring AMOT inhibition of YAP function. YAP–TEAD complex is promptly inactivated when germ layers are specified, and this event is needed to adjust PSC mechanical properties in response to physiological substrate stiffness. By providing evidence that YAP–TEAD1 complex targets key genes encoding for proteins involved in cytoskeleton dynamics, we suggest that substrate mechanics can direct PSC specification by influencing cytoskeleton arrangement and intracellular tension. We propose an aberrant activation of YAP–TEAD1 axis alters PSC potency by inhibiting cytoskeleton dynamics, thus paralyzing the changes in shape requested for the acquisition of the given phenotype.

## Introduction

During cell differentiation and organogenesis, cells encounter a rearrangement in their shape and size which is instrumental to the acquisition of their new identity [1]. This process requires the dynamic adjustment of the cytoskeleton and is guided by an interplay between biochemical and mechanical cues arising from the extracellular matrix (ECM) or provided by the neighbouring cells [2–5].

Gradients in intracellular tension within the embryo are thought to play a role in the differential regulation of Yes-associated protein (YAP) [6]. During foetal heart and liver development, YAP is critical to achieve the correct cell number [7]. Aberrant YAP expression or defects in mechanosensitive Hippo pathway lead to tissue overgrowth, organomegaly [7–9] and embryonic lethality [10].

YAP acts downstream of Hippo kinase network and integrates mechanical and biochemical signals arising from the ECM and the surrounding cells to shuttle to the nucleus and activate given genetic programmes, by interacting with cell- and stage-specific transcription factors [11–15]. Our group recently showed YAP co-transcriptional activity in breast cancer cells is triggered by cell spreading [16] and reinforces cell-matrix interaction by promoting focal adhesion (FA) assembly [17].

The cooperation of YAP with transcriptional regulators to maintain embryonic stem cell (ESC) pluripotent state has been recently questioned: the effects of its depletion are mild [18, 19], while elevated YAP levels favour adult cell reprogramming to pluripotency [20]. Its transient overexpression in somatic cells reverts their maturation to the state of tissue-specific progenitors [21].

Substrate mechanical cues regulate adult progenitor fate [22], and differentiated cell function [23–27] through YAP [14]. Whether YAP function is mechanically regulated in human embryos and pluripotent stem cells (PSCs) and if its co-transcriptional activity can be exploited to maintain their potency or drive their specification is still debated.

Here we demonstrate that undifferentiated PSCs display a sustained YAP–TEAD transcriptional activity, which is not sensitive to contact inhibition. Tampering with Tankyrase-AMOT axis restores contact inhibition of YAP nuclear shuttling. YAP–TEAD can be—instead—further stimulated by ECM stiffening to regulate PSC mechanical properties by controlling the expression of proteins involved in cytoskeleton stabilization. The fine tuning of YAP–TEAD-induced cell tension is required during PSC mesoderm specification in order to allow the timely rearrangement of the cytoskeleton these cells need to acquire a new identity.

## Materials and methods

### Cell culture, differentiation and drug treatments of human PSC lines

The human iPSC line DF 19-9-7T (iPSCs, karyotype: 46, XY) was purchased from WiCell (Madison, WI, USA). The STENF iPSC line was a gift from Prof. I. Koutna (Masaryk University, Brno, Czech Republic). The YAP knockout (YAP−/− or KO) and isogenic H9 (WT or CTR) human embryonic stem cell lines (hESCs) were a kind gift of Miguel Ramalho-Santos and Han Qin. Their generation and culture were described in [20]. The cells were maintained in an undifferentiated state by culturing them onto Matrigel Growth Factor Reduced (1:100 in DMEM/F12, Corning, NY, USA) in complete Essential 8™ Medium (E8, Thermo Fisher Scientific, Waltham, MA, USA) containing penicillin/streptomycin (0.5%, VWR).

Mesoderm and Cardiac differentiation followed the protocol of Lian et al. with slight modifications [19]. Briefly, PSC colonies were dissociated into single cells (TrypLE Select, Thermo Fisher Scientific) and re-seeded onto Matrigel-coated plates at 2.0 × 10^5^ cells/cm^2^ in complete medium with Rock Inhibitor Y27632 (2.5 μm, Selleck chemicals, Houston, TX, USA). The following day, the medium was replaced and then changed daily until the cells reached 100% confluence. At day 0, the medium was substituted with mesoderm induction medium consisting of RPMI 1640 (Sigma-Aldrich, St. Louis, MO, USA) supplemented with penicillin/streptomycin, l-glutamine (2 mm, Biowest, Riverside, MO, USA), B-27™ supplement minus insulin (1×, Thermo Fisher Scientific) and CHIR99021 (8 µm, Sigma-Aldrich). At day 2, the medium was replaced with RPMI/B-27 minus insulin (+B-27 − Ins) supplemented with IWP-2 (5 µm, Selleck chemicals). At day 4, the medium was substituted with RPMI + B-27 minus insulin and replaced every other day until the cells started beating (iPSC-CMs); at that time RPMI was supplemented with B-27 plus insulin (+B-27 + Ins) and changed every 2–3 days throughout differentiation to maintain the beating cells.

For trilineage differentiation assay, a Human Pluripotent Stem Cell Functional Identification Kit (R&D System, Minneapolis, MN, USA) was used. Briefly, undifferentiated iPSCs or hESCs (0.5 × 10^5^/cm^2^) were plated and after 48 h they were challenged with ectoderm, endoderm and mesoderm differentiation medium according to the manufacturer’s instructions.

For drug treatments, iPSCs, at day 0 of differentiation, were stimulated for 24 h Leptomycin B (20 nm, Sigma-Aldrich). After 24 h, the cells were processed for qPCR or immunofluorescence. hESCs were stimulated with jasplakinolide (50 nm, Thermo Fisher Scientific), WNT3A (1 nm, R&D System) for 24 h, and XAV939 (10 µm, Absource Diagnostic, Munich, Germany) for 48 h in undifferentiation medium for Atomic Force Microscopy (AFM) analysis or at day 0 of differentiation and then processed at day 2 for RT-qPCR. CAL51 and YAP−/− CAL51 cell lines were cultured as previously described [17]. All the cell lines used in the study have been tested for mycoplasma contamination regularly.

### Micropatterned cell culture

For micropatterned cell culture, CYTOOchips™ ARENA glass coverslips (CYTOO, Grenoble, France), with different sizes of round areas (140, 225, 500 and 1000 µm), were activated with Poly-L-Lysine Hydrobromide (40 μg Sigma-Aldrich) in distilled water (1 mL) for 2 h at room temperature and then treated with diluted Matrigel (1:100 in PBS) for 24 h at 37 °C according to the manufacturer’s instructions. After 24 h, undifferentiated PSCs were seeded (2 × 10^6^ cell/coverslip) onto the Matrigel-coated coverslips without letting the surfaces dry out throughout the cell seeding. After 48 h culture in complete medium, or medium supplemented with XAV939 the cells were analyzed by immunofluorescence. Detailed “Materials and methods” can be found in Supplemental materials section.

## Results

### YAP–TEAD1 axis controls PSCs mechanics independently of cell–cell contact

YAP nuclear activity is sensitive to substrate stiffness and negatively regulated by cell–cell interactions in numerous adult cell types [11, 12]. In such cells, YAP shuttling to the nucleus was observed on substrates stiffer than 0.5 [12] or 5 kPa [28].

**Fig. 1:**
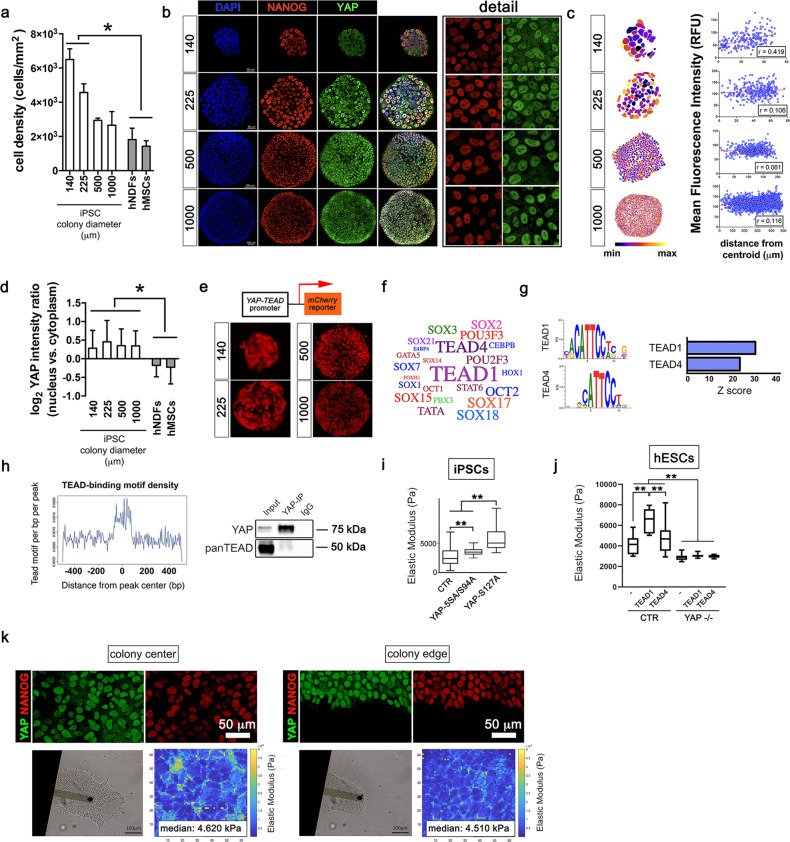
YAP–TEAD1 transcriptional activity controls pluripotent stem cell mechanical properties regardless of contact inhibition. **a** Barplot representation of the quantification of cell density in iPSCs grown onto micropatterned surfaces with the indicated diameters (μm), high density hMSCs and hNDFs. Values are expressed as means ± SD (*n* = 6, *P < 0.05, one-way ANOVA followed by Holm-Sidak’s multiple comparisons test). **b** Representative confocal images (*n* = 10) depicting YAP (green) and NANOG (red) expression in iPSCs grown onto micropatterns of the given diameters (μm). **c** Quantification of YAP distribution within the micropatterned colonies of controlled diameter as quantified by image analysis and expressed as the Pearson’s product moment correlation coefficient (*n*_(140 ⌠m)_ = 19; *n*_(225 ⌠m)_ = 9; *n*_(500 ⌠m)_ = 9; *n*_(1000 ⌠m)_ = 6). **d** Barplot representation of the quantification of YAP nucleus/cytoplasm intensity ratio in iPSCs grown onto micropatterned surfaces with the indicated diameters (μm), high-density hMSCs and hNDFs. Values are expressed as means ± SD (*n* = 6, *P < 0.05, one-way ANOVA followed by Holm-Sidak’s multiple comparisons test). **e** Representative confocal images of YAP–TEAD-mCherry reporter hESC line cultured onto micropatterns with the indicated diameters (μm) (*n* = 3). **f** wordcloud representation of the most significantly represented transcription factors known to bind the sequences identified as YAP targets by ChIP-seq analysis. Font size correlates inversely to −log10(*P* value). **g** Left: motif analysis identification of enriched YAP ChIP-seq peaks with the relative statistical significance. Right: barplot representation of enriched YAP ChIP-seq peaks with the relative statistical significance. **h** Left: graphical representation of TEAD-binding motif density within a 500-bp distance from YAP ChIP-seq peak. Right: western blot analysis for anti-YAP and -panTEAD antibodies in iPSCs immunoprecipitated for YAP endogenous protein. Input and IgG were used as positive and negative controls, respectively (*n* = 3). **i** Boxplot representation of the Elastic Modulus (or Young’s Modulus) of single iPSCs transfected with GFP (day 0) or co-transfected with GFP and either YAP-S127A or YAP-5SA/S94A mutants, as obtained by Atomic Force Microscope (AFM) analysis. Values are shown as median ± min/max (*n* = 12, **P < 0.01, Kruskal–Wallis test followed by post hoc Dunn’s test for multiple comparison). **j** Boxplot representation of the data obtained by analyzing the Elastic Modulus of single CTR or YAP−/− hESCs transfected with GFP (−) or co-transfected with GFP and either TEAD1 or TEAD4. Values are shown as median ± min/max (*n* = 12, **P < 0.01, Kruskal–Wallis test followed by post hoc Dunn’s test for multiple comparison). **k** Top: representative confocal images of NANOG (red) and YAP (green) expression at the centre or at the edge of hESC colonies (*n* = 3). Bottom: bright-field images of AFM cantilever contacting cells at hESC colony centre or edge and the respective Elastic modulus maps obtained from the measurement.

Human embryonic stem cell lines (hESCs) and induced pluripotent stem cells (iPSCs) grow in compact colonies at high cell density; while this condition is usually associated in somatic epithelial cells to YAP inactivation and nuclear exclusion, YAP remains predominantly expressed in PSC nuclei (Supplementary Fig. 1a). We cultured iPSCs onto micropatterned substrates that allow precise manipulation of colony size (diameter: 140, 225, 500 and 1000 μm) and cell density, and compared YAP subcellular localization to adult human mesenchymal stem cells (hMSCs) or dermal fibroblasts (hNDFs) grown at a similar density. iPSC density in micropatterned colonies correlated inversely with colony area (Fig. 1a), while YAP appeared mostly expressed in the nucleus and co-localized with pluripotency markers NANOG (Fig. 1b), OCT4 and β-CATENIN. (Supplementary Fig. 1b).

YAP intracellular localization was largely independent on the position of the cell within the iPSC colony, while being affected by cell density in hMSCs and hNDFs (Fig. 1c, d). We confirmed YAP–TEAD transcriptional activity (as measured by luciferase assay on 8xGTIIC-lux-transduced cells) was significantly reduced in dense culture of adult cells, consistent with YAP exclusion from the nucleus (Supplementary Fig. 1c). Instead, YAP–TEAD transcriptional activity in PSCs was homogeneous throughout the micropatterned colonies regardless the increasing density, as shown by hESC reporter line based on the expression of mCherry fluorescent tag under YAP–TEAD promoter (Fig. 1e) [16].

Our data indicate that PSCs show a sustained activation of YAP, raising the question of what is its function in this context. Thus, we pulled down endogenous YAP in iPSCs and performed chromatin immunoprecipitation (ChIP) followed by DNA sequencing (Seq) analysis. YAP ChIP-seq analysis yielded 5208 unique binding sites, mostly located in intergenic and intronic regions (Supplementary Fig. 1d, e, Supplementary Table 1). Bioinformatics analysis of the ChIP-seq data identified possible TFs interacting with YAP in PSCs, which were not previously described in adult cells (Fig. 1f), and detected TEAD1 and TEAD4 binding motifs as the most overrepresented in proximity of YAP binding sites (Fig. 1g). Indeed, the density of TEAD-binding motifs within 500-base pair (bp) distance of YAP ChIP-seq peaks and co-immunoprecipitation analysis confirmed the physical interaction between YAP and TEAD in iPSCs (Fig. 1h). TEAD transcription factor family is deemed responsible for ~78% YAP co-transcriptional regulation in adult cells [29]. Of note, TEAD proteins showed nuclear localization regardless of PSC density (Supplementary Fig. 1f).

Our group recently found YAP–TEAD controls adult cell mechanical properties by reinforcing their interaction with the ECM [17, 30]. We, hence, measured by AFM the stiffness of single iPSCs co-transfected either with YAP-S127A mutant (constitutively activating gene transcription through TEAD) or its transcriptionally active form unable to bind TEAD (YAP-5SA/S94A) [13], and GFP. Cells overexpressing TEAD-dependent mutant were significantly stiffer (5300 ± 3553 vs. 2466 ± 1666 kPa) than those transfected with the TEAD-independent and GFP control (1677 ± 1287 kPa). TEAD-independent mutant also induced a slight but significant increase in cell Young’s Modulus (Fig. 1i).

Given that TEAD1 and TEAD4—the two TEAD isoforms identified by our bioinformatics analysis—play distinct roles in development [31, 32], we asked which component of TEAD family was responsible for PSC stiffening following YAP overexpression. We ectopically expressed either TEAD1 or TEAD4 in CTR or in YAP−/− hESCs [33]. When compared to the GFP control (*E*_CTR-GFP_ = 4170.0 ± 891.7 Pa), TEAD1 overexpression significantly increased cell Young’s Modulus (*E*_CTR-TEAD1_ = 6466.08 ± 1131.70 Pa), while TEAD4 had a modest, non-significant effect (*E*_CTR-TEAD4_ = 4310.21 ± 919.63 Pa). No effect was found when TEAD1 or TEAD4 were transfected in YAP−/− hESCs (*E*_KO-GFP_ = 2894.0 ± 308.9 Pa; *E*_KO-TEAD1_ = 3056.2 ± 206.9 Pa; E_KO-TEAD4_ = 2988.1 ± 150.6 Pa) (Fig. 1j). We next probed the elasticity of cells located in different positions within the colony and found no significant changes in the stiffness of cells at the centre (*E*_centre_ = 4 620 ± 3 622 Pa) or at the edge (*E*_edge_ = 4510 ± 3380 Pa) of the colony (Fig. 1k). In contrast, the exclusion of YAP from the nuclei of confluent adult cells correlated with a significant reduction in cell mechanics (*E*_confluent_ = 8078 ± 4275 Pa vs. *E*_sparse_ = 17,726 ± 4279 Pa) (Supplementary Fig. 1g).

The results suggest that YAP–TEAD1 activity supports the mechanical properties of PSCs independently of cell–cell contacts.

### Contact inhibition of YAP–TEAD1 transcriptional activity is restored by AMOT downstream of Tankyrase

Next, we tried to unveil the molecular mechanism involved in YAP restriction in confluent adult/differentiated cells, which is missing in PSCs.

To address this issue, we used PSC-derived cardiomyocytes (Supplementary Fig. 2a**)** as a model of differentiated cells able to control YAP localization [34] (Supplementary Fig. 2b**)**. We looked for YAP negative upstream regulators exclusively expressed in the differentiated state. As expected, the differentiation process was highlighted by the downregulation of pluripotency genes and the concomitant upregulation of early and late cardiac markers (Supplementary Fig. 2c–e) and confirmed Hippo was one of the pathways regulated during the process (Supplementary Fig. 2f). Among the negative upstream regulators of YAP, we found Angiomotin (*AMOT*), Angiomotin-like protein 2 (*AMOTL2*), Dachsous Cadherin-Related 1 (*DCHS1*) and FAT Atypical Cadherin 4 (*FAT4*) consistently upregulated during cardiac differentiation (Supplementary Fig. 2g). The list of genes significantly regulated in day 0 hESCs as compared to day 15 hESC-derived cardiomyocytes is shown in Supplementary Table 2.

**Fig. 2:**
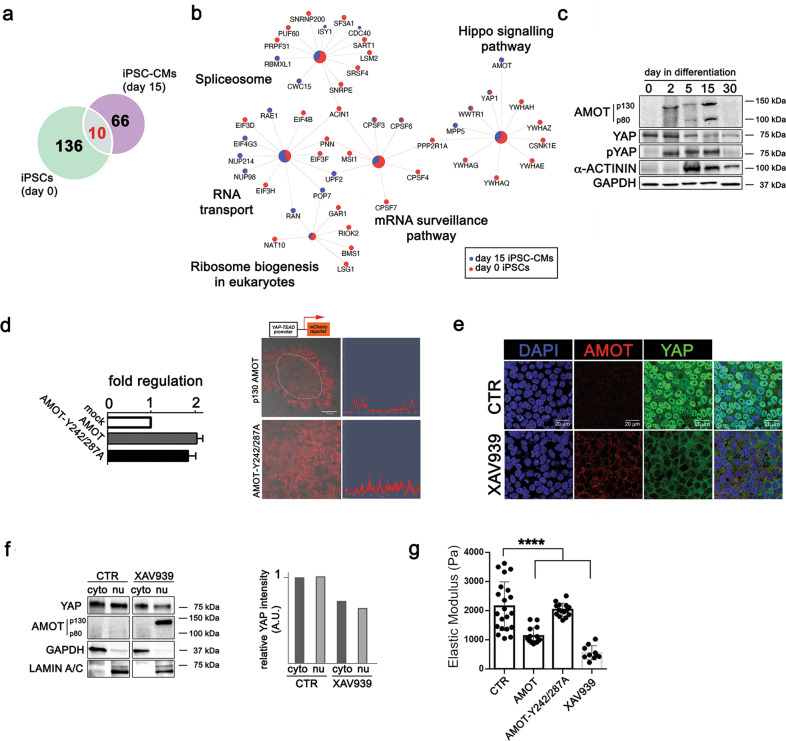
Contact inhibition of YAP nuclear localization in PSCs is restored by Tankyrase-p130-AMOT. **a** Venn diagram representation of the common pool of YAP-interacting proteins in iPSCs (day 0, *n* = 3) and iPSC-derived cardiomyocytes (iPSC-CMs, day 15, *n* = 3) as obtained by mass spectrometry analysis of the endogenous YAP protein. **b** Graphical representation of YAP interactome in day 15 iPSC-CMs (blue) versus day 0 iPSCs (red). Mass spectrometry results were fed to Cytoscape and analyzed by KEGG database. The size of the origin of the nodes is proportional to the *P* value (*P* < 0.01; Kappa score = 0.3). The fractions of the colours are weighted on the number of proteins belonging to the given node at day 0 or day 15. **c** Western blot analysis of the indicated proteins in iPSCs at the indicated days of cardiac differentiation. Alpha sarcomeric actinin (α-ACTININ) was used as a marker of differentiated cardiomyocytes. GAPDH was used for total protein loading normalization. The blots are representative of three independent experiments. **d** Left: barplot representation of AMOT RNA fold regulation in H9 hESCs transduced with AMOT-p130 (AMOT), AMOT-p130-Y242/287A or empty vector (mock). The data are indicated as average ± SD *n* = 2. Right: representative fluorescence-brightfield superimposed image and relative quantification of YAP–TEAD-mCherry hESCs transduced with either AMOT-p130 (AMOT) or AMOT-p130-Y242/287A vectors. Image analysis of mCherry fluorescence within PSC colony is shown. **e** Representative confocal images depicting AMOT (red) and YAP (green) expression in H9 hESCs treated or not with XAV939 for 48 h (*n* = 3). (f) Top: western blot analysis of the indicated proteins in cytoplasm (cyto) or nucleus (nu) of iPSCs treated or not with XAV939 for 48 h. GAPDH and LAMIN A/C were used to normalize cytoplasmic and nuclear proteins, respectively. Bottom: quantification of YAP protein levels in cytoplasm (cyto) or nucleus (nu) of iPSCs treated or not with XAV939 for 48 h. The blots are representative of three independent experiments. **g** Barplot representation of the Elastic Modulus of hESCs (CTR) transfected with either AMOT-p130 (AMOT) or AMOT-p130-Y242/287A, or treated with XAV939 for 48 h as obtained by AFM analysis (*n* = 12, ****P < 0.0001, Kruskal–Wallis test followed by post hoc Dunn’s test for multiple comparisons).

We confirmed by RT-qPCR the upregulation of *AMOT*, *AMOTL2*, *FAT4* and *DCSH1*, together with Angiomotin-like protein 1 (*AMOTL1*) and Neurofibromin 2 (NF2) in both day 15 and day 30 cardiomyocytes, as compared to undifferentiated cells (Supplementary Fig. 3a, b). In parallel, we immunoprecipitated endogenous YAP in undifferentiated iPSCs (day 0) and iPSC-derived beating cardiomyocytes (day 15) and performed mass spectrometry (MS) analysis of YAP-interacting proteins. The differential analysis identified 146 proteins interacting with YAP at day 0, and 76 at day 15. Only ten of these interactors were common to both stages (Fig. 2a). We focused on the 66 unique YAP interactors in day 15 iPSC-cardiomyocytes and pointed at AMOT as the main YAP negative upstream regulator being absent in undifferentiated cells (Fig. 2b). AMOTL1 and AMOTL2 were instead found also among YAP interactors in undifferentiated iPSCs, while FAT4, NF2 and DCHS1 were not detected in either condition (Supplementary Table 3), likely due to indirect or weak interactions. A schematics of YAP interactors in iPSCs and iPSC-derived cardiomyocytes is indicated in Supplementary Fig. 4.

P130-AMOT isoform was recently found to restrict YAP nuclear presence in adult cells [35] and during PSC neural differentiation [36]. We detected by western blot an accumulation of AMOT-p130 and p80 protein isoforms at day 2, 5 and 15 of cardiac differentiation. This event was paralleled by the YAP phosphorylation (Fig. 2c). Next, we asked whether AMOT would be able to restore YAP sensitivity to cell contacts in PSCs and affect their mechanical properties. Hence, we stably expressed p130-AMOT in YAP–TEAD-mCherry hESCs and obtained a twofold mRNA overexpression, similar to the threefold increase observed during differentiation (Supplementary Fig. 3b). We also expressed p130-AMOT-Y242/287A mutant (1.8-fold), which is not able to bind YAP [36]. By checking mCherry fluorescence, we noticed p130-AMOT re-expression—but not the mutant—was able to reduce YAP–TEAD transcriptional activity in confluent hESCs (Fig. 2d). To provide independent evidence for AMOT role, we took advantage of the observation that Tankyrase regulates p130-AMOT degradation, such that Tankyrase inhibitors result in AMOT-dependent YAP inhibition [37]. We therefore treated hESCs with Tankyrase inhibitor XAV939 and found increased AMOT protein expression and YAP shuttling to the cytoplasm (Fig. 2e). In fact, XAV939 treatment caused an accumulation of p130-AMOT in the nuclei of PSCs and a reduction in both nuclear and cytoplasmic YAP (Fig. 2f).

Finally, we measured the stiffness of hESCs transduced with p130-AMOT, p130-AMOT-Y242/287A mutant, or exposed to XAV939. PSCs in which p130-AMOT inhibitory function was restored had significantly reduced stiffness, in keeping with inhibited YAP activity, while expression of p130-AMOT-Y242/287A mutant had no effects (*E*_CTR_: 2148 ± 837.3 Pa; *E*_p130-AMOT_: 1131 ± 280.7 Pa; *E*_p130-AMOT-Y242/287A_ = 2030 ± 224.7 Pa; *E*_XAV939_ = 553.6 ± 240.2 Pa; Fig. 2g). Collectively, these results suggest that increased degradation of AMOT in PSCs accounts, at least in part, for the reduced ability of cell–cell contacts to regulate YAP.

### YAP–TEAD1 control over cytoskeleton-related genes mediates PSC stiffening in response to substrate rigidity

**Fig. 3:**
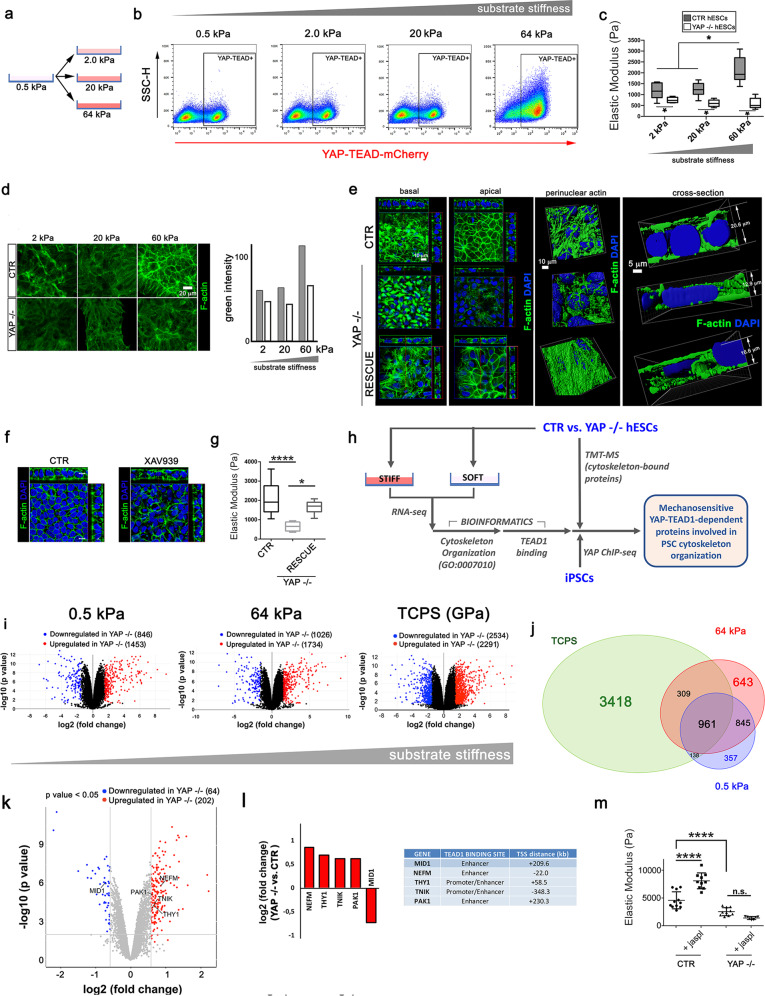
YAP–TEAD1 acts downstream of substrate stiffness to transcriptionally control cytoskeleton-related genes and PSC mechanics. **a** Graphical representation of the experimental setup used to assess pluripotent stem cell (PSC) response to changes in physiological substrate stiffness. YAP–TEAD-mCherry hESC reporter cells were cultured onto soft surface (0.5 kPa) and then moved to surfaces with increasing stiffness (2, 20 and 64 kPa). **b** Representative FACS plots depicting mCherry fluorescence in YAP–TEAD-mCherry hESC reporter cells cultured for 48 h on substrates with physiological stiffness (*n* = 3). **c** Boxplot representation of the Elastic Modulus of CTR and YAP−/− hESCs grown onto substrates with increasing stiffness (2, 20 and 60 kPa). The values were obtained by AFM and are expressed as Pascal (Pa) (*n* = 12, *P < 0.05, one-way ANOVA test followed by Holm-Sidak’s test for multiple comparison). **d** Left: representative confocal images depicting F-actin (green) cytoskeleton arrangement in isogenic (CTR) and YAP−/− hESCs (YAP−/−) grown onto substrates with increasing physiological stiffness. Right: barplot representation of the intensity of green channel (F-actin) in isogenic (CTR) and YAP−/− hESCs (YAP−/−) grown onto substrates with increasing physiological stiffness (*n* = 3). **e** Left: orthogonal sections from Z-stack confocal images showing the basal (left) and apical (right) distribution of F-actin in CTR, YAP−/− and YAP −/− hESCs in which YAP has been re-expressed (RESCUE). F-actin is stained with Phalloidin (green) and nuclei counterstained with DAPI (blue). Side views show sagittal sections of the monolayered cells. Right: 3D Z-stack reconstruction and cross-sectional view of the perinuclear actin of CTR, YAP−/− and RESCUE hESCs (*n* = 3). The images were obtained by IMARIS software after staining with Phalloidin (F-actin, green) and DAPI (nuclei, blue). **f** Orthogonal sections from Z-stack confocal images showing F-actin organization (green) in CTR and XAV939-treated hESCs for 48 h. Side views show sagittal sections of the monolayered cells. F-actin was stained with Phalloidin (green) and nuclei were counterstained with DAPI. **g** Boxplot representation of the elastic modulus of CTR, YAP−/− and RESCUE hESCs (*n* = 12, ****P < 0.0001; *P < 0.05, Kruskal–Wallis test followed by post hoc Dunn’s test for multiple comparisons). **h** Schematic representation of the strategy followed to discover proteins involved in cytoskeleton organization which are regulated by substrate stiffness through YAP in pluripotent stem cells (PSCs). **i** Volcano plot representation of differentially regulated genes in CTR versus YAP−/− hESCs grown on substrates with physiological (0.5 and 64 kPa) and tissue culture polystyrene (TCPS). (*n* = 3, *P* < 0.05, log2 Fc < |0.58|). **j** Venn diagram representation of differentially regulated genes in CTR versus YAP−/− hESCs grown on substrates with physiological (0.5 and 64 kPa) and tissue culture polystyrene (TCPS). **k** Venn diagram representation of PSC YAP *bona fide* targets that have an annotation for cytoskeleton organization (GO: 0007010) and found dysregulated onto substrate with controlled stiffness (0.5 and 64 kPa) and Tissue culture polystyrene (TCPS). **l** Volcano plot representation of cytoskeleton-bound proteins significantly regulated in YAP−/− compared to CTR hESCs, as identified by TMT Mass Spectrometry. (*n* = 5, *P* < 0.05, log2 Fc > |0.58|). **l** Left: barplot representation of cytoskeleton-bound proteins significantly regulated in YAP−/− compared to CTR hESCs, as identified by TMT mass spectrometry, that were defined as YAP *bona fide* targets with cytoskeleton annotation (GO:0007010). Right: identification of TEAD1-binding sites in selected YAP targets with cytoskeleton organization annotation. **m** Dotplot representation of elastic modulus in CTR and YAP−/− hESCs treated or not with F-actin polymerizing agent jasplakinolide for 24 h as obtained by AFM analysis. (*n* = 12, ****P < 0.0001, Kruskal–Wallis test followed by post hoc Dunn’s test for multiple comparisons).

We next quantified mCherry fluorescence in YAP–TEAD-mCherry hESCs cultured onto soft PDMS-coated surface (0.5 kPa) that were switched to either 2, 20 or 64 kPa (Fig. 3a). YAP–TEAD transcriptional activity was not significantly affected by switching cells from 0.5 to 2 or 20 kPa surface (mCherry_0.5kPa_ = 45.2 ± 3.9%; mCherry_2kPa_ = 41.6 ± 1.1%; mCherry_20kPa_ = 40.5 ± 1.0%, respectively). On the contrary, a consistent increase in mCherry-positive cells was found on 64 kPa (mCherry_64kPa_ 75.5 ± 3.8%, Fig. 3b). These data suggested the mechanical activation of YAP–TEAD1 complex in PSCs occurs on substrates with very high stiffness (*E* > 20 kPa) compared to somatic cells where the threshold is around 0.5–5 kPa [29]. We therefore asked whether this delayed increase in YAP–TEAD transcriptional activity induced by substrate stiffening altered PSC mechanical properties.

hESCs cultured on 60 kPa were significantly more rigid than those cultured on softer substrates, which were not significantly different among themselves (*E*_64kPa_ = 2112.26 ± 601.54; *E*_20kPa_ = 1239.70 ± 298.57; *E*_2kPa_ = 1170.90 ± 350.33). Moreover, YAP−/− hESCs were significantly softer than the isogenic counterpart and unable to respond to substrate stiffening (*E*_64kPa_ = 593.74 ± 242.27 Pa; *E*_20kPa_ = 599.43 ± 149.29 Pa; *E*_2kPa_ = 753.52 ± 121.26 Pa), thus indicating that high physiological substrate stiffness controls PSC mechanical properties through YAP–TEAD1 (Fig. 3c).

Cell nanoscale stiffness correlates with the accumulation of F-actin bundles during breast cancer dissemination [38]. We stained F-actin in control hESCs cultured on substrates with increasing stiffness and found higher degree of actin fibre organization paralleled increased cell stiffness in control cells. On the contrary, no changes were detected in the actin of YAP mutant cells in response to substrate stiffening (Fig. 3d).

In cells lacking YAP cortical actin was lost and the apico-basal polarity of the adherent monolayer altered in comparison to control cells (Fig. 3e). Also, perinuclear actin cap was deranged and the overall thickness of the cell monolayer reduced. The reintroduction of YAP in knockout hESCs was, instead, sufficient to restore F-actin organization, similar to the isogenic control (Fig. 3e, Supplementary videos 1–3). This condition was phenocopied by treating PSCs with Tankyrase inhibitor XAV939 (Fig. 3f), which is able to hinder YAP–TEAD1 axis and hESC stiffness (see Fig. 2e, f). As expected, YAP reintroduction in knockout cells also restored their elastic modulus (*E*_CTR_ = 2148 ± 873.3 Pa; *E*_YAP−/−_ = 648.7 ± 227.6 Pa; *E*_RESCUE_ = 1642 ± 317.2 Pa) (Fig. 3g).

We next looked for YAP–TEAD1 transcriptional targets accounting for PSC mechanical properties (Fig. 3h). YAP controls the transcription of genes encoding for proteins involved in F-actin polymerization in murine heart [39]. We performed differential RNA-seq analysis on YAP−/− and isogenic hESCs on substrates with increasing stiffness, which proved to have limited (soft: 0.5 kPa) or high (stiff: 64 kPa, TCPS) effects on YAP–TEAD transcriptional activity and PSC cytoskeleton arrangement. As expected, the number of genes regulated by substrate stiffness through YAP increased steadily with stiffness (0.5 kPa: 2299; 64 kPa: 2796; TCPS: 4825) (Fig. 3i), with TCPS and 64 kPa sharing a higher number of genes in comparison to 0.5 kPa (Fig. 3j). Next, we looked for mechanically activated YAP transcriptional targets having an annotation for cytoskeleton organization (GO:0007010), which were physically bound by YAP in iPSC ChIP-seq analysis on TCPS. We selected those genes that were found significantly regulated in YAP−/− hESCs RNA-seq on stiff (TCPS or 64 kPa) as compared to soft (0.5 kPa) surfaces. Detailed data coming from the ChIP-seq and RNA-seq analysis are reported in Supplementary Tables 1, 4 and in Supplementary Fig. 5a, b.

We matched these results with differences in cytoskeleton-bound proteins in YAP−/− hESCs by quantitative TMT-MS and uncovered 266 cytoskeletal proteins differentially regulated (>1.5-fold) in cells depleted for YAP (Fig. 3k) out of 6014 total proteins (Supplementary Table 5).

Among these, we found few well-known controllers of cytoskeleton integrity: TRAF2 and NCK-interacting protein kinase (TNIK) [39], P21 (RAC1) Activated Kinase (PAK1) [40], Thy-1 Cell Surface Antigen (THY1) [41, 42], and Neurofilament Medium [43] were found upregulated by 1.55, 1.54, 1.62 and 1.82 folds, respectively. Midline 1 (MID1) [44] was, instead, found downregulated by 1.7 times in the absence of YAP. We confirmed by bioinformatics that all the genes identified harbour a binding site for TEAD1 transcription factor, either in the enhancer or in the promoter (Fig. 3l).

Altogether, we hypothesized that changes in the expression of such key cytoskeleton proteins would make YAP-depleted PSCs unable to promptly assemble cortical actin and respond to substrate stiffening by increasing their mechanics. To confirm this hypothesis, we forced actin polymerization in YAP-depleted and isogenic hESCs by jasplakinolide [45], and mapped cell stiffness by AFM. Control cells treated with jasplakinolide became significantly stiffer than the untreated control. Instead, the effect could not be phenocopied in cells lacking YAP (Fig. 3m), since they were not induced to stiffen by the pharmacological agonist of actin polymerization.

Altogether, these results demonstrated that YAP–TEAD1 determines PSC stiffening in response to substrate mechanical cues by transcriptionally controlling the expression of key cytoskeleton-related genes.

### YAP–TEAD1-driven cell stiffening correlates with intracellular tension and determines cell contractility

**Fig. 4:**
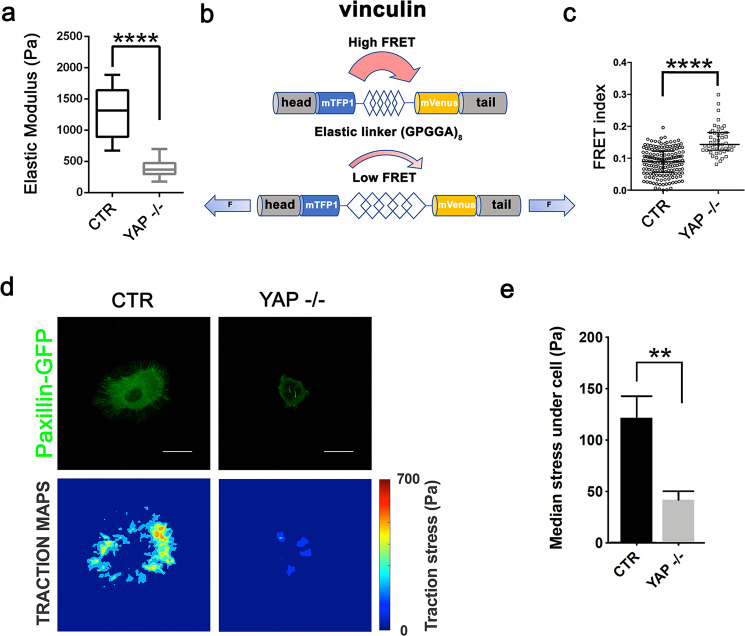
YAP–TEAD1-driven cell stiffening correlates with intracellular tension and determines cell contractile force. **a** Boxplot representation of the elastic modulus of isogenic CAL51 and YAP−/− CAL51 cells as obtained by AFM. The values are expressed in Pascal (Pa). (*n* = 12, ****P < 0.0001, Mann-Whitney test). **b** Schematic representation of the genetically encoded Förster Resonance Energy Transfer (FRET) sensor based on vinculin tension. **c** Dotplot representation of FRET index in isogenic CAL51 and YAP−/− CAL51 cells as determined by FRET for vinculin tension sensor (*n* = 6, ****P < 0.0001, Mann-Whitney test). **d** Representative traction force maps for paxillin-GFP transfected CTR and YAP−/− CAL51 cells. Stress values are expressed in Pascal (Pa). **e** Barplot representation of traction forces exerted by CTR and YAP−/− CAL51 cells. The values are represented as median ± SD (*n* = 6, **P < 0.01, Mann–Whitney test).

Next, we investigated whether cell mechanical properties correlated with the magnitude of tension propagated across cell FAs through F-actin cytoskeleton, like previously suggested [46]. To this aim, we transiently transfected YAP−/− [17] and isogenic Cal51 cells with a Förster Resonance Energy Transfer (FRET) vinculin tension sensor [47]. YAP−/− cells displayed a significantly lower elastic modulus than the isogenic control (Fig. 4a) and a higher FRET index, which correlates inversely with the mechanical tension applied on vinculin at FAs (Fig. 4b, c).

The intracellular tension generated throughout the cytoskeletal network is transmitted through the FAs to the ECM, and can be measured by traction force microscopy [48]. We seeded Paxillin-GFP YAP−/− Cal51 cells and their isogenic control onto poly-acrylamide gels (15 kPa) embedded with fluorescent microbeads (Fig. 4d). We calculated the median stress generated by single cells by computing microbead displacement and found YAP-depleted cells developed a lower force than the control (Fig. 4e). These results indicated that soft YAP-depleted cells display reduced intracellular tension throughout the cytoskeleton, which reflects in a limited ability to exert force on the ECM.

### YAP–TEAD1-guided cytoskeleton remodelling is needed for mesoderm specification

**Fig. 5:**
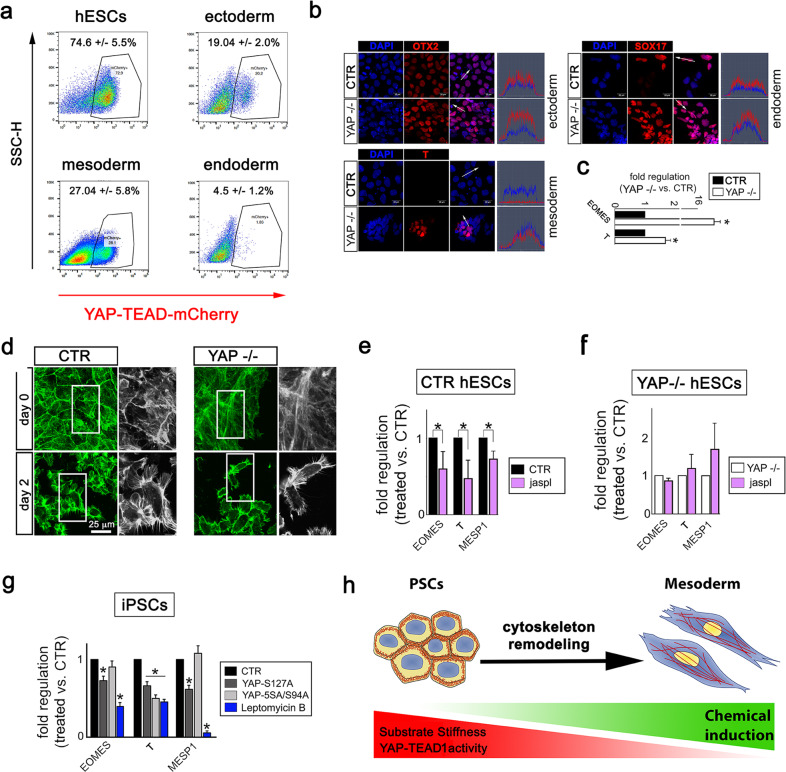
YAP–TEAD1 sustained activation hampers the remodelling of cytoskeleton required for PSC mesoderm specification. **a** Representative FACS plots depicting mCherry fluorescence in YAP–TEAD-mCherry PSC reporter cells cultured for 48 h in control (hESCs) or in mesoderm, ectoderm or endoderm media. The data are presented as percentage ± SD (*n* = 3). **b** Representative confocal images for the indicated lineage-specific markers (mesoderm, ectoderm and endoderm, in red) as detected in CTR or YAP−/− hESCs after 3 days stimulation with lineage-specific differentiation medium. Nuclei were counterstained with DAPI (blue) and image analysis is shown to quantify the intensity of the fluorescent signals (*n* = 3). **c** Barplot representation of the expression of the indicated mesoderm genes in CTR or YAP−/− hESCs induced to mesoderm specification for 2 days (*n* = 4, *P < 0.05, one-way ANOVA test followed by post hoc Holm-Sidaks test for multiple comparisons). The data are shown as fold regulation ± SD in YAP−/− as compared to CTR hESCs. **d** Representative confocal images depicting F-actin organization (green) in CTR and YAP−/− cells in the undifferentiated state (day 0) or induced to mesoderm specification (day 2) (*n* = 3). **e** Barplot representation of the expression of the indicated mesoderm genes in CTR hESCs cultured in mesoderm differentiation medium, supplemented with jasplakinolide (24 h) for 2 days. The data are shown as fold regulation ± SD in treated as compared to untreated cells (*n* = 3, *P < 0.05, ANOVA test followed by post hoc Holm-Sidaks test for multiple comparisons). **f** Barplot representation of the expression of the indicated mesoderm genes in YAP−/− hESCs cultured in mesoderm differentiation medium, supplemented with jasplakinolide for 3 days. The data are shown as fold regulation ± SD in treated as compared to untreated cells (*n* = 3, no significance found after ANOVA test followed by post hoc Holm-Sidaks test for multiple comparisons). **g** Barplot representation of the expression of the indicated mesoderm genes in iPSCs transfected with either YAP-S127A or YAP-5SA-S94A, or treated with nucleus export blocker Leptomycin B and induced to mesoderm differentiation for 3 days. The data are shown as fold regulation ± SD in treated cells as compared to CTR (*n* = 4, *P < 0.05, one-way ANOVA test followed by post hoc Holm-Sidaks test for multiple comparisons). **h** Schematic representation of the model proposed for YAP–TEAD1 interference with cytoskeleton remodelling during PSC mesoderm specification.

The control of cytoskeleton integrity is crucial for PSC [49], MSC [1, 11, 50] and keratinocyte differentiation [51]. We asked whether changes in cytoskeleton arrangement, as those described in cells depleted of YAP–TEAD1 transcriptional activity, would be instrumental to PSC specification. We noticed YAP–TEAD-mCherry reporter hESCs exposed to trilineage specification underwent a consistent drop in mCherry signal regardless of the lineage (ectoderm: 19.04 ± 2.0%; mesoderm: 27.04 ± 5.8%; endoderm: 4.5 ± 1.2%) as compared to the undifferentiated control (74.6 ± 5.5%, Fig. 5a). Moreover, we found YAP mutant cells were more prone to acquire mesoderm and endoderm markers as compared to their isogenic counterpart when appropriately stimulated, while no difference in ectoderm specification could be detected (Fig. 5b). YAP involvement in mesoderm lineage specification was confirmed by RT-qPCR showing an accumulation of EOMES and T RNAs in YAP−/− cells (Fig. 5c). Therefore, we focused on the role of YAP–TEAD1 on cytoskeleton remodelling and intracellular tension during PSC mesoderm specification.

We compared F-actin arrangement in CTR and YAP−/− cells before (day 0) and during mesoderm induction (day 2). Cortical actin—a typical feature of undifferentiated control cells found deranged in YAP-depleted hESCs—was substituted by stress fibers during mesoderm specification. Cells induced to mesoderm appeared significantly smaller than their pluripotent counterpart and displayed a distinct elongated shape (Fig. 5d).

Next, we triggered mesoderm specification in isogenic and YAP−/− hESCs exposed to jasplakinolide and found the ability of isogenic hESCs to acquire mesoderm markers *EOMES, T* and *MESP1* was significantly affected when actin remodelling was impaired (Fig. 5e). Again, no change could be found in YAP−/− cell ability to undergo mesoderm specification (Fig. 5f).

In addition, we transfected iPSCs with either YAP-S127A or YAP-5SA/S94A, which differentially regulate intracellular tension and cell stiffness (see Fig. 1j), and induced mesoderm specification. While stiffer YAP-S127A-transfected PSCs showed a significant reduction in the expression of mesoderm genes *EOMES, T* and *MESP1*, cells transfected with YAP-5SA/S94A showed only reduced *T* (Fig. 5j and Supplementary Fig. 7a). This result was phenocopied by Leptomycin B treatment of mesoderm-induced cells, which causes YAP nuclear retention (Fig. 5g, Supplementary Fig. 7b).

These results indicated that sustained activation of YAP–TEAD1 transcriptional axis hinders PSC mesoderm specification by interfering with the fine tuning of cytoskeleton remodelling required for the process (Fig. 5h).

## Discussion

The role of YAP protein in PSC maintenance and differentiation is still debated [18–21]. Here we demonstrate that the transcriptional activity of YAP–TEAD complex is sustained in PSCs and insensitive to contact inhibition, while being promptly repressed during cell specification. Similar to adult cells [17], YAP transcriptionally drives PSC stiffening, mainly by interacting with TEAD1 transcription factor. TEAD-independent transcription also induces a mild but significant stiffening of PSCs, very likely because genes involved in cell stiffening harbourbinding domains for transcription factors other than TEAD.

As a result of the inability of YAP protein to perceive cell–cell contacts, pluripotent cell colonies are mechanically homogeneous. This property distinguishes PSCs from adult cells, which adjust their rigidity by responding to cell density so that cells packed in dense colonies are softer than sparse ones.

The response of YAP–TEAD to substrate mechanics also marks a difference between PSCs and adult cells. The transcription complex displays a delayed sensitivity to physiological changes in ECM mechanics in PSCs, with a threshold for YAP nuclear shuttling being above 20 kPa. This threshold is way higher than the one described for adult cells [12, 28]. The reduced sensitivity of PSCs to substrate stiffening and cell–cell contacts could be considered as a shield embryonic cells put in place against mechanical stress.

Given the unique response of YAP to mechanical cues in PSCs, we looked for exclusive upstream regulators that were absent in the pluripotent cells and active in differentiated ones. YAP–TEAD transcriptional activity is progressively reduced during cardiac maturation, so that cardiomyocytes are able to restrict YAP nuclear shuttling to the nucleus [15]. By exploiting PSC differentiation to cardiomyocytes, we identified Tankyrase-AMOT axis as the missing link impeding YAP inhibition downstream of cell–cell interaction in PSCs. Tankyrase keeps AMOT levels low in PSCs, so that the protein cannot physically bind to YAP and hamper its function. The ectopic expression of p130-AMOT, the isoform able to physically interact with YAP, or Tankyrase pharmacological inhibition were indeed sufficient to reduce YAP activity in PSCs, thus driving cell softening. A similar role for AMOT was described in PSC neural commitment [36], while inhibitors of Tankyrase have been recently proposed to negatively regulate YAP by stabilizing AMOT in tumor cells [37]. Nonetheless, these inhibitors have a wide spectrum of activities, and thus the possibility that other pathways (i.e., Wnt, Akt) are also involved in YAP inhibition cannot be excluded.

Breast cancer cell stiffening has been lately correlated with the accumulation of F-actin bundles [38]. In independent investigations, YAP itself has been associated with tumor spreading and a poor prognosis [52]. The protein also contributes to gastric cancer cell cytoskeleton remodelling [53]. A direct effect on genes encoding for cytoskeleton proteins was suggested for YAP in murine heart [54].

We found PSC stiffening in response to physiological surface rigidity depends on YAP–TEAD1 transcriptional activation of a handful of genes encoding for proteins involved in the control of cytoskeleton dynamics. Among these proteins, YAP–TEAD1 represses the transcription of *TNIK*, which disrupts F-actin structure [40] and contribute to the activity of ARP2/3, the complex controlling the nanoscale architecture of cortical actin in ESCs [55, 56]. Together with the dysregulation of *THY1, PAK1* and *MID1* genes [40, 44], *TNIK* upregulation in YAP-depleted cells most likely explains why their cytoskeleton appears less organized and they develop less intracellular tension and force.

Together with the well-established evidence that YAP activation responds to F-actin integrity, our data indicate the existence of a positive loop fuelling cytoskeleton stability through YAP–TEAD1 transcriptional activity. This loop has been suggested to involve both YAP and its paralog protein TAZ [57]. Despite the two proteins are known to act in redundancy in different adult cell types [11], we found YAP depletion brought to a slight decrease in TAZ levels on very stiff substrates (TCPS). Further experiments are needed to explore the role of YAP paralog protein in pluripotent cells.

F-actin cytoskeleton dynamics is of utmost importance for cell differentiation [49–51]. Mesoderm cells are, in fact, significantly smaller than PSCs and their elongated shape is dictated by stress fibers, rather than by cortical actin, which confers a more regular appearence to undifferentiated PSCs.

**Fig. 6:**
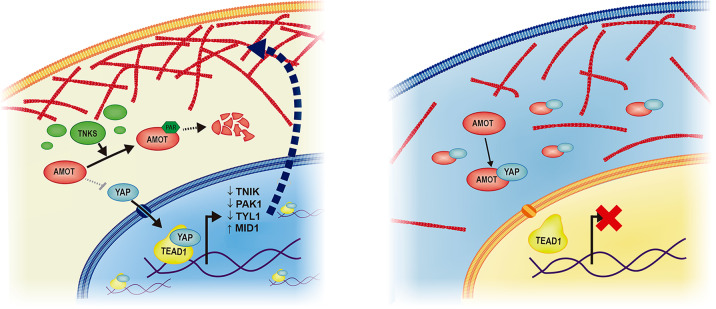
Proposed model for Tankyrase-mediated regulation of cytoskeleton stability and cell mechanics through AMOT and YAP–TEAD1 during PSC specification. Left: pluripotent stem cells (PSCs) growing into high confluence colonies display high Tankyrase activity, in turn keeping the levels of AMOT low, independently of cell–cell interactions. Under such circumstances, YAP is free to shuttle to the nucleus and modulate, among the others, the expression of genes involved in actin stability, like *TNIK*, *PAK1, THY1 and MID1*. Right: during mesoderm specification, Tankyrase activity is low, AMOT protein expression increases, so that in confluent cells, YAP can be restricted to the cytoplasm in response to cell–cell contact. In these conditions, cytoskeleton remodelling can occur.

We show actin cytoskeleton remodelling during PSC specification requires YAP–TEAD1 deactivation and can be hindered by enhancing cell intracellular tension and rigidity, by either forcing YAP nuclear presence or by artificially inhibiting F-actin remodelling (Fig. 6).

These lines of evidence further our understanding of the molecular pathways underlying the mechanical regulation of PSC phenotype and function, and identify a specific management of the mechanosensing apparatus in PSCs in response to ECM stiffness and cell–cell interaction.

## Supplementary information

Supplementary Information

Supplementary Table 1

Supplementary Table 2

Supplementary Table 3

Supplementary Table 4

Supplementary Table 5

Supplementary Table 6

Supplementary Table 7

Supplementary video 1

Supplementary video 2

Supplementary video 3
