# Supplementary material for: YAP–TEAD1 control of cytoskeleton dynamics and intracellular tension guides human pluripotent stem cell mesoderm specification
Source: Cell Death Differ. 2020 Oct 28;28(4):1193–207. doi: 10.1038/s41418-020-00643-5 (PMC8027678; doi:10.1038/s41418-020-00643-5)
Supplement: Supplementary file 1 — Supplementary Information [file 41418_2020_643_MOESM1_ESM.docx]

**Supplementary Information**

**Materials and Methods**

**Cell Transfection, Lentiviral Infection and FACS analysis**

Cell transfection and co-transfection were performed in Opti-MEM™ (Thermo Fisher Scientific) medium using FuGENE® HD Transfection Reagent (2.5 μl/μg of DNA, Promega, Madison, WI, USA) and the following plasmids: p2xFLAGhYAP1-S127A (Addgene#17790, gift from Marius Sudol); pCMV-Flag-YAP-5SA/S94A (Addgene#33103, gift from Kun-Liang Guan); pmaxFPTM-Green-N vector (VDF-1012, Amaxa); pPGS-3HA-TEAD1 (Addgene#33055, gift from Kunliang Guan); Myc-TEAD4 (Addgene#24638, gift from Kunliang Guan) and pmaxFPTM-Green-N vector (VDF-1012, Amaxa Byosistems, Cologne, Germany).

For lentiviral infection, SureENTRY Transduction Reagent (6 µg/mL, QIAGEN, Hilden, Germany) was used to deliver lentiviral vectors. iPSCs were infected with YAP/8SA (FLAG-YAP2 (8SA)-pcw107, #Addgene64637, gift from David Sabatini & Kris Wood) lentiviral particles and subjected to puromycin selection for 2 weeks before using them. For overexpressing YAP, lentiviral particles containing YAP construct (pGAMA-YAP, Addgene#74942, gift from Miguel Ramalho-Santos) or control (mock/empty vector) were delivered to YAP-/- hESCs or CAL51 cells. YAP-TEAD (pLL3.7 K122 -ires-GFP-TEAD-responsive-H2B mCherry reporter, Addgene #128327, gift from Yutaka Hata) lentiviral particles were used to infect CTR hESCs. This cell line (YAP-TEAD-mCherry hESCs) was seeded onto CYTOOchips™ ARENA glass coverslips or plates with different stiffness. Forty-eight hours later, the cells were either imaged by confocal microscope (from ARENA) or collected by TrypLE Select (from plates), analyzed by Beckman Coulter MoFlow Astrios Cell Sorter (Beckman Coulter Life Sciences, Brea, Ca, USA) and plots prepared using FlowJo software V10 (Tree Star, Ashland, OR, USA). The same cell line was also used for trilineage differentiation assay and analyzed by Cell Sorter.

**Luciferase Assay**

ASC52telo, hTERT immortalized adipose-derived mesenchymal stem cells (hMSCs, ATCC® SCRC-4000™, Manassas, VA, USA) and human Normal Dermal Fibroblasts (hNDFs, a gift from Prof. I. Koutna, Masaryk University, CZ), were cultured in DMEM containing 4.5 g/l Glucose (DMEM high Glucose, Lonza) supplemented with 10% fetal bovine serum (Sigma-Aldrich), L-glutamine (2 mm) and penicillin/streptomycin (100 U/ml), and then seeded in a 96 MW plate (2.0x10^4^ cell/well). After 48h, the cells were co-transfected with an 8xGTIIC-lux plasmid (Addgene#34615, a gift from Stefano Piccolo) in which a YAP/TEAD-responsive synthetic promoter drives luciferase expression, and the pRL-TK Renilla (Promega) control at a 1:1 ratio (200 ng/well).

Luciferase assay was performed on cells 24 h after treatments using the Dual-Glo® Luciferase Assay System kit (Promega) according to the manufacturer’s protocol and analyzed on a Berthold CENTRO LB 960 Microplate Luminometer (Berthold Technologies GmbH, Bad Wildbad, Germany). The measured luciferase activities were normalized to the Renilla activity.

**Cell culture on plates with different stiffness.**

PDMS-coated plates were prepared by mixing different ratios of Sylgard 184 and Sylgard 527 (Sigma-Aldrich) to obtain stiffness ranging from 2 kPa up to 60 kPa (1). The resulting mixtures were degassed, poured in desired vessels and polymerized at 65°C overnight.

Stiffness of material was measured using custom made device controlled by the proprietary LinuxCNC software running on the real-time kernel (2). Samples were loaded by monotonic compression and release which enabled measurement in 0% strain. For cell culture experiments, PDMS was coated with Matrigel™ identically to normal tissue culture plates.

CytoSoft® 6-well plates with a thin layer of anhydride-functionalized silicone with different stiffness (0.5 and 64 kPa) were purchased from Advanced BioMatrix (Carlsbad, CA, USA) and used according to the manufacturer’s instructions.

The surface of the gels was pre-coated with Matrigel (1:100 in DMEM/F12) before seeding hESCs. After 48h from seeding, the cells were collected for RT-qPCR, RNA–seq and FACS analysis.

**Atomic Force Microscopy (AFM) preparation**

Cell samples were probed using a BioAFM microscope JPK NanoWizard 3 (JPK, Berlin, Germany). Samples in 34 mm Petri dish (TPP, Trasadingen, Switzerland) were placed under a 100x100x15 μm scanning head (X-Y-Z axis respectively) and kept in cell culture medium or moved to calcium-free Tyrode medium at 37°C by a Petri dish heater (JPK) before the measurement. Where indicated, calcium chloride was replenished in Tyrode solution 15 minutes before the measurement as to reach 0.4- or 1.8-mm final concentration. Where indicated, jasplakinolide was added to the culture medium to reach the final concentration of 50 nm 1 hour before the measurement. The sample and the AFM probe were visualized by an inverted optical microscope Olympus IX-81 (Olympus, Tokyo, Japan) equipped with epifluorescence system for GFP visualization. Non-coated silicon nitride AFM cantilevers Hydra 2R-100N or NITRA TALL B (AppNano, Mountain View, CA, USA) equipped with pyramidal silicon tip (half-angle 18°) were used for all the experiments, according to the height of the sample. The cantilever was calibrated in cell medium, before each experiment, calculating the system deflection sensitivity by single force-distance curve (FDC) on freshly peeled mica. The probe spring constant was then estimated by the thermal noise method. Cantilever stiffness ranged between 11 and 43 pn nm^−1^ for different days of experiments.

**Force Mapping**

The AFM setting was identical for all the force mapping procedure. Force maps were recorded in cell medium, which was exchanged with fresh one each hour. The peak applied force was 1.0 nN (relative to baseline value), Z-length 15.0 μm and speed of curve recording 30 μm s^−1^. The FDCs were recorded with data sample rate of 5 kHz. The force mapping procedure was performed as pointwise recording of FDCs on a 100 × 100 μm area including several PSCs. The network resolution was 16 × 16, 32 × 32 or 64 × 64 points based on the resolution needed.

**AFM** **Data Analysis**

Elasticity values (Young modulus) were calculated in JPK Data Processing software by fitting each FDC by Sneddon equation with Bilodeau’s correction (3):

$$F\left( \delta\right)=\frac{0.7453 E \alpha}{\pi(1-\upsilon^{2})}\delta^{2}$$

Where *F* is the measured force, *E* is Young’s modulus, ν is Poisson ration (0.5 for incompressible materials), δ is tip-sample separation and α is half-angle to edge of pyramidal tip. Resulted Young’s modulus maps were exported to be further analyzed. The elasticity maps obtained from measurements were processed in open source SPM analysis software Gwyddion to extract geometrically coherent text tables of elasticity values. Subsequently, data were filtered by a Matlab (Mathworks, Natick, MA, USA) script to select the portion of data belonging to the biological sample and export them.

All the processed values were imported in Graphpad Prism 6.0 for statistical analysis. Datasets first underwent normality test (Kolmorogov-Smirnof). When some datasets presented non-gaussian distribution, groups were compared by Kruskal-Wallis non-parametric test, with Dunn’s post-hoc test for multiple comparisons. Statistical significance was accepted for *P*-values smaller than 0.05. Data are presented as median ± IQR (25%-75% interquartile distance), with whiskers chosen by Tukey’s rule for boxplots.

**Quantitative Real-Time PCR (RT-qPCR)**

Total RNA was extracted using a High Pure RNA Isolation Kit (Roche, Base, Switzerland) according to the manufacturer’s instructions. Reverse transcription (1 μg of RNA) was performed using Transcription First Strand cDNA Synthesis Kit (Roche) and real-time qPCR was carried out in triplicate, using a LightCycler 480 SYBR Green I Master Kit (Roche) and run on a LightCycler 480 Real-Time PCR System (Roche). The expression level of individual genes was determined by ∆Ct method relative to the expression of the housekeeping gene GAPDH. Primer sequences are provided in Supplementary Table 6. The results include the heatmaps of quantification cycles (Ct) and the graphs of the means and SD values of the fold regulation values obtained by analyzing independently three samples per experimental condition.

Pathway RT² Profiler PCR Array (PAHS-172Z, QIAGEN) was used to analyze the expression of 84 genes involved in the Hippo signal transduction cascade in iPSCs at different stages of cardiac differentiation (day 0, day 15 and day 30). RT–PCR reaction was run on the LightCycler 480 Real-Time PCR System using the following cycling parameters: 1 cycle at 95°C for 10 min; 45 cycles at 95°C for 15 s and 60°C for 1 min. The internal panel of housekeeping genes set from the manufacturer was used for normalization of expression levels of individual genes and PCR-array data analyzed by online resources provided on the manufacturer’s website (http:/www.qiagen.com). The results include the heatmaps of quantification cycles (Ct) and the graphs of the means and SD values of the fold regulation values obtained by analyzing independently three samples per experimental condition.

**RNA Sequencing (RNA-seq) and Data Analysis**

Library was prepared using NEBNext® Ultra™ II Directional RNA Library Prep Kit for Illumina® with NEBNext® Poly(A) mRNA Magnetic Isolation Module and NEBNext® Multiplex Oligos for Illumina® (Dual Index Primers Set 1). Kits were employed according to manufacturers’ protocol, input for library preparation was 200-300 ng total RNA.

Sequencing was done on Illumina NextSeq 500 using NextSeq 500/550 High Output v2 kit (75 cycles). We have done single-end 75bp sequencing in multiple sequencing runs until all samples had at least 30 million passing filter reads. Fastq files were generated using bcl2fastq software without any trimming. The quality of the raw sequencing data was assessed using FastQC (https://www.bioinformatics.babraham.ac.uk/projects/fastqc/) and aligned to the hg38 reference genome using the TopHat2 aligner (4). Raw gene counts were obtained by calculating reads mapping to exons and summarized by genes using reference gene annotation (Ensembl 90; Homo sapiens GRCh38.p10, GTF) by HTSeq (5). Differential gene expression was performed using DESeq2 bioconductor package. Genes were considered as differentially expressed when the Benjamini-Hochberg adjusted *P* value ≤ 0.05 and log2 fold-change (log2FC) ≥ 1.5. Biological-term classification and the enrichment analysis of gene clusters was performed using clusterProfiler (6). All computations were performed with R 3.5.1 (R Core Team 2018), and the R packages gplots and GOplot were used (7).

The Gene Ontology (GO) categories for germ layer specification were downloaded from AmiGO 2 repository (<https://amigo.geneontology.org>).

**ChIP-seq and Data Analysis**

Undifferentiated iPSCs were seeded onto 100 mm dishes (four plates for each sample) in complete medium for 4 days. Chromatin was immunoprecipitated from three technical replicates using a ChIPgrade anti-YAP antibody (Cell Signaling Technologies, Danvers, MA, USA; see Supplementary Table 7) and following the manufacturer’s protocol (Pierce™ Agarose ChIP Kit, Thermo Fisher Scientific). A control ChIP was performed using rabbit immunoglobulins (IgG). The samples were eluted in 30 μL eluting buffer and stored at -80 °C before analysis. For library preparation, the size distribution of each ChIP DNA sample was assessed by running a 1 µL aliquot on Agilent High Sensitivity DNA chip using an Agilent Technologies 2100 Bioanalyzer (Agilent Technologies, Santa Clara, CA, USA). The concentration of each DNA sample was determined using a high sensitivity Quant-iT™ dsDNA Assay Kit and a Qubit Fluorometer (Thermo Fisher Scientific). Purified ChIP DNA (10 µg) was used as the starting material for sequencing libraries preparation. Indexed libraries were prepared using a TruSeq ChIP Sample Prep Kit (Illumina Inc., San Diego, CA, USA). The libraries were sequenced (single read, 1x50 cycles) at a concentration of 10 pm/lane on a HiSeq 2500 (Illumina Inc.).

Data analysis was performed by Genomix4Life S.r.l. (Salerno, Italy). The raw sequence files generated (.fastq) underwent quality control analysis using FastQC (http://www.bioinformatics.babraham.ac.uk). The reads were aligned to the human genome (assembly hg19) using bowtie (8), allowing up to one mismatch and considering uniquely mappable reads. The reads of replicates and corresponding input samples were merged for peaks calling as previously described (9). ChIP-Seq peaks were identified and analysed using HOMER Motif Database (-F: 2.0, -L: 2.0 and -C: 1.0) with a false discovery rate < 0.01 (Ref. 10). The assignment of YAP peaks to target genes was obtained using the web tool ChIPSeek (11).

Through this step, it was possible to assign peaks to the transcription start site (by default defined from - 1 kb to + 100 bp), transcription termination site (by default defined from -100 bp to + 1 kb), Exon (Coding), 5’-untranslated region (UTR) Exon, 3’ UTR Exon, Intronic or Intergenic regions. As some annotations overlap, the following order of priority was chosen for the assignment:

- Transcription start site (by default defined from - 1 kb to + 100 bp)
- Transcription termination site (by default defined from -100 bp to + 1 kb)
- CDS exons
- 5’ UTR exons
- 3’ UTR exons
- **CpG islands
- **Repeats
- Introns
- Intergenic

Over-represented sequence motifs were defined according to motif descriptors in the JASPAR database and computed using PScan-ChIP (12). The following parameters were set:

- Organism: Homo Sapiens Assembly: hg19
- Background: Mixed
- Descriptors: Jaspar 2016

Nucleotide best occurrence was calculated by WebLogo (http://weblogo.berkeley.edu/) by running a Report Best Occurrences analysis on any given transcription factor.

Functional enrichment analysis was performed using Ingenuity® Pathway Analysis (IPA®, QIAGEN, www.qiagen.com/ingenuity) with the following parameters:

- Reference set: Ingenuity Knowledge Base (Genes Only)
- Relationship to include: Direct and Indirect
- Filter: Consider only molecules and/or relationship where (species = Human) AND (confidence = Experimentally Observed).

All raw and final processed RNA-seq and ChIP-seq data have been deposited on ArrayExpress (<https://www.ebi.ac.uk/arrayexpress/>). The RNA-seq and ChIP-seq data can be accessed by using the following accession numbers, respectively: **E-MTAB-7619** and **E-MTAB-7620**.

**Western Blotting**

For total protein extraction, the cells were lysed in RIPA buffer (Merck Millipore, Burlington, MA, USA) supplemented with a protease and phosphatase inhibitor cocktail (1%, Sigma-Aldrich) on ice for 20 min and then centrifuged at 16,000 x g for 10 min at 4°C. Protein concentration was determined by BCA method (Thermo Fisher Scientific) with a spectrophotometer (Multiskan™ GO, Thermo Fisher Scientific) set at 562 nm using bovine serum albumin as standards. Protein samples (7 µg each) were loaded in 10% polyacrylamide gels prepared using TGX™ FastCast™ Acrylamide Solutions (Bio-Rad, Hercules, CA, USA) and run at 100 V. The proteins were transferred to a polyvinylidene difluoride membrane (PVDF, Bio-Rad) using the Trans-Blot Turbo transfer system (Bio-Rad). Membranes were blocked with 5% BSA (Santa Cruz Biotechnology, Dallas, TX, USA) in TBST, incubated with diluted primary antibody in 5% BSA in TBST at 4°C with rotation overnight and then probed with the appropriate secondary HRP-conjugated antibody (Sigma-Aldrich) at room temperature for 1 h. A ChemiDoc imaging system (Bio-Rad) was used to detect chemiluminescence. The list of antibodies and their dilutions are detailed in Supplementary Table 7.

**Protein Co-Immunoprecipitation**

For immunoprecipitation of YAP-binding proteins, iPSCs (n=3) were seeded in 100 mm dishes (4 plates for each sample) and lysed after 4-5 days of culture. Total proteins were obtained by scraping the cells in the presence of lysis buffer (50 mmTris, pH 7.4, 150 mmNaCl, 1 mm EDTA) supplemented with 0,5% TERGITOL™ solution Type NP-40 (Sigma-Aldrich) and a protease and phosphatase inhibitor cocktail for 25 min on ice. Lysates were cleared by centrifugation at 16,000 x g for 10 min at 4 °C and equal amounts of proteins for each sample (500 μg) were incubated with YAP antibody (Sigma-Aldrich) or isotype control IgG and Protein G Sepharose 4 Fast Flow (GE Healthcare Life Sciences, Chicago, IL, USA) overnight under rotation at 4°C. The immunoprecipitates were then washed three times with lysis buffer (without protease/phosphatase inhibitors) followed by two washes in lysis buffer (without detergent and protease/phosphatase inhibitors) at 4 °C under rotation. The samples were then loaded onto a 10% Mini-PROTEAN® TGX™ Precast Gel (Bio-Rad) and immunoblotting was performed as described above.

**LC-MS/MS, Protein Identification and Data analysis**

Following immunoprecipitation washes, bead-bound protein complexes (n=7, for each condition) were digested directly on the sepharose beads by addition of 0.75 µl (1µg/µl) of trypsin (sequencing grade, Promega) in 50mm NaHCO_3_ buffer. The beads were gently tapped to ensure even suspension of the trypsin solution and then were incubated at 37°C with mild agitation for 2 h. The beads were then mixed by vortexing to dissociate the complex from the beads and then the partially digested complex was separated from beads and incubated at 37°C overnight (16 h) without agitation. The resulting peptides were extracted into LC-MS vials by 2.5% formic acid in 50% acetonitrile (ACN) and 100% ACN with added polyethylene glycol (20 000; final concentration 0.001%) as described previously (17), and then concentrated in a SpeedVac concentrator (Thermo Fisher Scientific). Liquid Chromatography Mass Spectrometry (LC-MS/MS) analyses of peptide mixtures were performed using a RSLCnano system connected to an Orbitrap Elite hybrid spectrometer (Thermo Fisher Scientific). Prior to LC separation, tryptic digests were online concentrated and desalted using a trapping column (100 μm × 30 mm) filled with 3.5-μm X-Bridge BEH 130 C18 sorbent (Waters Corporation, Milford, MA, USA). After washing the trapping column with 0.1% formic acid, the peptides were eluted (flow 300 nl/min) onto an analytical column (Acclaim Pepmap100 C18, 3 μm particles, 75 μm × 500 mm; Thermo Fisher Scientific) using a 100 min nonlinear gradient program (1-56% of mobile phase B; mobile phase A: 0.1% formic acid in water; mobile phase B: 0.1% FA in 80% ACN). The trapping column and the analytical column were equilibrated prior to sample injection into the sample loop. The analytical column outlet was directly connected to a Digital PicoView 550 (New Objective, Woburn, MA, USA) ion source with PicoTip emitter SilicaTip (New Objective; FS360-20-15-N-20-C12). An ABIRD (Active Background Ion Reduction Device) was installed. MS data were acquired in a data-dependent strategy selecting up to the top 10 precursors based on precursor abundance in the survey scan (350-2 000 m/z). The resolution of the survey scan was 60,000 (400 m/z) with a target value of 1×10^6^ ions, one microscan and a maximum injection time of 200 msec. HCD MS/MS spectra were acquired with a target value of 50,000 and a resolution of 15,000 (400 m/z). The maximum injection time for MS/MS was 500 msec. Dynamic exclusion was enabled for 45 s after one MS/MS spectra acquisition and early expiration was disabled. The isolation window for MS/MS fragmentation was set to 2 m/z. The mass spectrometric RAW data files were analyzed using Proteome Discoverer software (Thermo Fisher Scientific; version 1.4) with in-house Mascot (Matrix Science, Boston, MA, USA; version 2.6) and Sequest search engines. MS/MS ion searches were first performed against the modified cRAP database (based on http://www.thegpm.org/crap/; 111 sequences in total) containing protein contaminants such as keratin and trypsin. MS/MS spectra assigned by the Mascot search engine to any cRAP protein peptide with a Mascot ion score > 30 were excluded from the next database searches. Final database searches were performed against the UniProtKB proteome database for Homo sapiens (taxonomy ID 9606). The database was downloaded on 2017-01-17 and the number of proteins was 21,031. cRAP database was searched in parallel. Mass tolerance for peptides and MS/MS fragments were 10 ppm and 0.05 Da, respectively. Oxidation of methionine, deamidation (N, Q) and acetylation (protein N-terminus) as optional modifications and two enzymes miss cleavages were set for all searches. Percolator was used for post-processing of the search results. Only peptides with a q-value < 0.01, rank 1 and with at least six amino acids were considered. Proteins matching the same set of peptides were reported as protein groups. Proteins within protein groups were reported only if they had at least one unique peptide. Label-free quantification using protein area calculation in Proteome Discoverer™ Software (Thermo Fisher Scientific) was used (“top 3 protein quantification”) (14). Protein group reports from all individual samples were combined into a single supergroup report where each supergroup is a list of proteins reported within a single protein group in at least a single sample report. Medium-confidence protein-protein interactions were obtained from the STRING database (https://string-db.org/). Interaction partners that could not be unambiguously identified were merged into one node (denoted by multiple gene names in a single node). In order to keep the visualization simple, a maximum of seven gene ontology (GO) terms or protein families were selected based on specific P-values. In iPSC-CMs the thresholds were as follows: *P* < 0.034 for protein families, *P* < 0.001 for biological process, *P* < 0.01 for both molecular function and cellular component. In undifferentiated iPSCs the thresholds were as follows: *P* < 0.05 for protein families and molecular function, *P* < 0.01 for biological processes and cellular components.

**Cytoskeleton protein extraction, LC-MS/MS and Data Analysis**

Undifferentiated YAP -/- hESCs (n=5) and their isogenic control (n=5) were cultured in 100 mm culture dishes for 48 hours. After this time, cytoskeleton proteins were extracted using Subcellular Protein Fractionation Kit (Thermo Fisher Scientific) following the manufacturer’s specifications. The samples were digested with trypsin using the filter-aided sample preparation (FASP) method (19). Briefly, similar amounts from each sample were reduced with 100 mm dithiothreitol at 56°C for 30 min, transferred to 30 kDa MWCO Pall Nanosep centrifugation filters (Sigma-Aldrich), washed several times with 8 m urea and once with digestion buffer (DB, 0.5% sodium deoxycholate in 50 mm triethylammonium bicarbonate prior to alkylation with 10 mm methyl methanethiosulfonate in digestion buffer for 20 min in room temperature. Digestions were performed by addition of 0.3 µg Pierce MS grade Trypsin (Thermo Fisher Scientific) in DB and incubated overnight at 37°C. An additional portion of 0.3 µg trypsin was added and incubated for another three hours. Peptides were collected by centrifugation and labelled using TMT 10-plex isobaric mass tagging reagents (Thermo Fisher Scientific) according to the manufacturer instructions. Labelled samples were combined and sodium deoxycholate was removed by acidification with 10% TFA. The TMT-labelled sample was fractionated by basic reversed-phase chromatography (bRP-LC) on a Dionex Ultimate 3000 UPLC system (Thermo Fisher Scientific). Peptide separation was performed using a linear gradient from 3% to 40% solvent B. Solvent A was 10mm ammonium formate buffer (pH 10.0) and solvent B was 90% acetonitrile (ACN), 10% solvent A. The column used was a reversed-phase XBridge BEH C18 column (3.5 µm, 3.0 x 150 mm, Waters Corporation). The sample was fractionated into 40 primary fractions and concatenated into 10 final fractions. The samples were dried and reconstituted in 3% ACN, 0.2% formic acid (FA) for LC-MS analysis.

Each fraction was analyzed on a Q Exactive HF mass spectrometer interfaced with an Easy-nLC1200 liquid chromatography system (both Thermo Fisher Scientific). Peptides were trapped on an Acclaim Pepmap 100 C18 trap column (100 μm x 2 cm, particle size 5 μm (Thermo Fisher Scientific) and separated on an in-house packed analytical column (75 μm x 30 cm, particle size 3 μm, Reprosil-Pur C18, Dr. Maisch). The gradient used was from 5% to 37% B over 68 min and from 37% to 48% B over 8 min followed by an increase to 100% B for 4 min and staying on 100% B for 10 min at a flow of 300 nL/min. Solvent A was 0.2% FA in water and solvent B was 80% ACN, 0.2% FA. The MS-instrument was operated in data-dependent mode where the MS scans were performed at a resolution of 60,000 and an m/z range from 400-1600. The 10 most intense ions were isolated using a 0.7 Da isolation window and fragmented using normalized collision energy of 33. MS2 scans were recorded at a resolution of 60,000, charge states 2 to 5 were selected for fragmentation and dynamic exclusion was set to 30 s with 10 ppm tolerance. The fixed first mass feature was used and set to 110 m/z.

The raw-data files of the TMT fractions were merged for identification and relative quantification using Proteome Discoverer version 2.4 (Thermo Fisher Scientific Scientific). The data was searched against the Homo sapiens Swissprot Database (version June 2019, Swiss Institute of Bioinformatics, Switzerland) using Mascot 2.5 (Matrix Science) with a precursor mass tolerance of 5 ppm and fragment mass tolerance of 30 mmu. The enzyme used was set to trypsin allowing 1 missed cleavage. Oxidation of methionine was set to variable and methylthio on cystein to static modification. TMT10-label modifications of peptide N-terminus and lysine were selected. TMT reporter ions were identified in the MS2 HCD spectra with 3 mmu mass tolerance. The quantification values were normalized on total peptide amount. Only peptides unique for a given protein were considered for quantification.

**Immunofluorescence**

Adherent cells were fixed in 4% PFA for 15 min at room temperature, permeabilized with 0.2% Triton X-100 (Sigma-Aldrich) for 5 min and blocked in 5% donkey serum (Sigma-Aldrich) and 2.5% BSA in DPBS solution for 1 h at room temperature. After incubation with primary antibodies (see Supplementary Table 7) for 2 h at room temperature, cells were washed three times with DPBS and stained with the appropriate Alexa fluorochrome-conjugated secondary antibodies (1:500 in DPBS, Thermo Fisher Scientific). The nuclei were counterstained with 4’,6’-diamidino-2-phenylindole (DAPI, Sigma-Aldrich). The samples were embedded in ProLong™ Diamond Antifade Mountant (Thermo Fisher Scientific) and visualized under a Zeiss LSM 780 confocal microscope (Zeiss, Oberkochen, Germany).

**Förster resonance energy transfer (FRET) microscopy**

To evaluate cellular forces at single focal adhesion (FA) sites, CTR and YAP-/- CAL51 cells were transfected with vinculin tension-sensor plasmid (Vinculin TS, Addgene#26019, gift from Martin Schwartz) using Lipofectamine 3000 (Thermo Fisher Scientific) and following the manufacturer’s protocol. After selection by neomycin resistance for 7 days, transfected cells were seeded on fibronectin-coated glass-bottom imaging dishes (µ-Dish, Ibidi) in complete FluoBrite DMEM (Thermo Fisher Scientific). After 24 h, spectral FRET was performed on a Nikon A1R+ confocal microscope (Nikon, Tokyo, Japan) equipped with an incubator (37 °C, 5% CO_2_) and a 32-channel spectral detector (470 to 650 nm, 2x binning), using a 457 nm excitation line and a 60x NA1.4 oil immersion objective. FRET index was calculated on a pixel basis from spectrally unmixed donor and acceptor signals (20), and values were averaged over single FAs.

**Traction Force Microscopy (TFM)**

TFM was conducted on polyacrylamide (PAA) gels synthesized by slightly modifying Plotnikov et al. (17). Typical gel formulation was as follows: 300 μL of methylacrylamide (40% solution Bio-Rad), 75 μL of N, N’ methylenebisacrylamide (2% solution, Bio-Rad), 5 μL of ammonium persulfate (10% aq., freshly prepared), 1.5 μL of N,N,N’,N’-tetramethylethylenediamine (Merck), 10 μL of red-fluorescent latex nanobeads (100 nm, 2% solids, Thermo Fisher Scientific), DIW q.s. 1 mL. Sixty μL aliquots were sandwiched between aldehyde-functionalized glass coverslips (40x22 mm, #1.5 thickness; activated by oxygen plasma, followed by vapor-phase silanization with (3-aminopropyl)trimethoxysilane, and exposed to GA (25% vapors) and hydrophobic glass slides, leading to coverslip-supported PAA substrates with a thickness around 70 µm and a shear modulus G = 15 kPa. Sulfo-SANPAH (1 mg/mL, Merck) coupling agent was grafted to PAA gel surface by UV crosslinking (365 nm, 900 mJ/cm^2^). After washing, activated PAA gels were coated with human fibronectin (20 μg/mL in PBS, Thermo Fisher Scientific) to promote cell adhesion.

CTR, YAP-/- CAL51 and YAP overexpressing CAL51 cells were infected with GFP-Paxillin lentiviral particles (pLV[Exp]-Neo-EF1A>hPXN3xGGGGS:EGFP, VectorBuilder, Chicago, IL, USA) by using Polybrene (5 μg/mL, Santa Cruz Biotechnology). After selection by neomycin resistance for 1 week, cells were seeded at low density (500 cells/cm^2^) onto PAA substrates. After 4 hours, slides were mounted into a confocal imaging chamber (RC30, Warner Instruments, Hamden, CT, USA).

TFM was performed on a Nikon A1R+ confocal microscope equipped with an incubator (37 °C, 5% CO_2_). Single cells were acquired at their FA focal plane with a 60x NA1.4 oil immersion objective, while simultaneously recording the underlying nanobead pattern. Reference micrographs (unstressed gel) were acquired in the same ROIs following trypsinization with 2.5% trypsin (Thermo Fisher Scientific) for 10 min. Datasets were analyzed by TFM package in MATLAB (18), using an L1-regularization method.

**Image Analysis**

We established an image analysis workflow for the quantification of YAP nuclear levels on confocal micrographs using ImageJ (19), and the MorphoLibJ plugin collection (20). The nuclear channel (DAPI) was pre-processed with a median filter to reduce noise and smoothen nuclei contours. Nuclei were identified using the Morphological Segmentation plugin, which includes a watershed algorithm to separate touching objects based on convexity. Using the regions of interest (ROIs) deriving from nuclear segmentation, intensity measurements were performed on the YAP channel (FITC); mean fluorescence intensity (MFI) levels associated to each nucleus were calculated and represented as a heat map (color limits were set to span the range of the data values). Considering the distance of each nucleus from the centroid of the cell aggregate, the radial distribution of nuclear YAP intensities was also calculated. iPSC density on micropatterned surfaces with controlled diameter was calculated by counting DAPI stained nuclei in ImageJ.

**Quantification and Statistical Analysis**

All the results are generated from two or three independent experiments performed in triplicate and the data presented as mean ± standard deviation SD, if not stated otherwise.

Data are presented as mean ± S. D. or median ± min/max as indicated in each figure panel. Sample sizes were based on previously published experiments, in which statistical differences were identified. Homoscedasticity and normality were tested using Barlett’s and Lilliesford tests, respectively, in order to choose between parametric and non-parametric statistical tests. For two-datasets comparison, statistical significance was calculated with t-test or Mann-Whitney tests, for parametric and non-parametric, respectively. For multiple comparisons, statistical significance was calculated using one-way ANOVA test followed by by post hoc Tukey’s test when parametric assumptions were met, while Kruskal-Wallis test followed by post hoc Dunn’s test was used otherwise. Bonferroni correction was applied for multiple comparisons .Specific test applied to each dataset is stated in the figure legend. Statistical differences were considered as positive when P < 0.05 or P < 0.01.

The statistical analysis for cell density on micropatterned colonies was performed by one-way ANOVA followed by Holm-Sidak’s multiple comparisons test. The number of colonies counted were as follows: n_(140 μm)_ = 19; n_(225 μm)_ = 9; n_(500 μm)_ = 9; n_(1000 μm)_ = 6. The association between radial position in the colony and nuclear YAP intensity was tested by Pearson’s product moment correlation coefficient. The contribution of TEAD to YAP DNA binding activity to genes belonging to cardiac-specific gene ontology (G) categories was quantified by using UpSetR (21).

**Supplementary Figures**

**
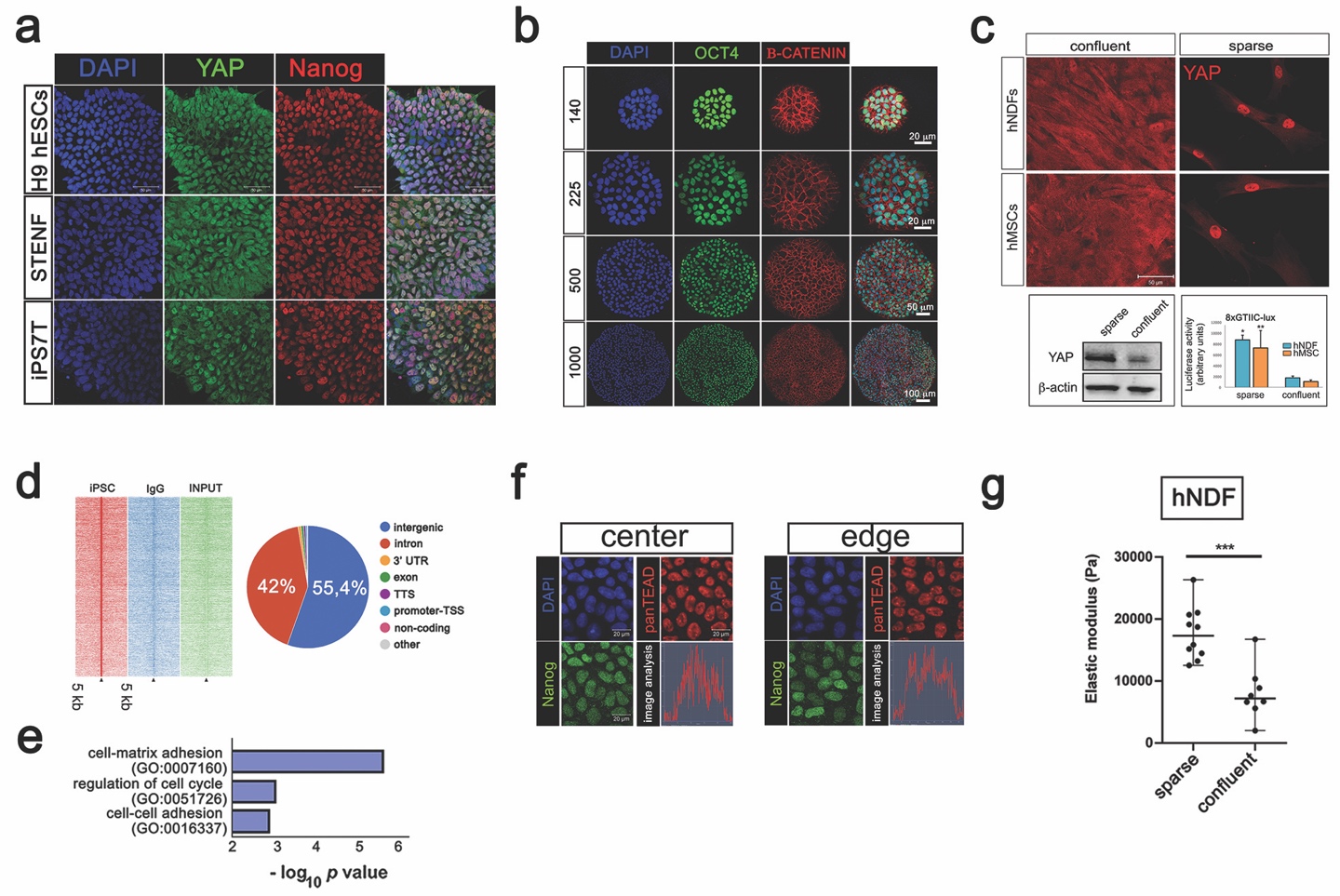
**

**Supplementary Figure 1. YAP expression and function in pluripotent and adult cells**. a) representative confocal images of YAP and Nanog expression in H9 human embryonic stem cells (hESCs), and STENF or iPS7T induced pluripotent stem cell (iPSCs) lines. b) Representative confocal images of micropatterned iPSC colonies of controlled diameter (140, 225, 500, 1000 μm) and stained for OCT4 (green) and BETA-CATENIN (red). c) ***Top:*** Representative staining of YAP localization (red) in confluent and sparse cultures of human normal dermal fibroblasts (hNDFs) and human mesenchymal stem cells (hMSCs); ***Bottom:*** Western blot quantification of YAP protein expression in confluent and sparse cultures and quantification of YAP-TEAD transcriptional activity in confluent and sparse cell cultures of hNDFs (right bottom). BETA-ACTIN was used for total protein loading normalization. *n*=3. **P* < 0.05, ***P* < 0.01. d) ***Left:*** Heatmap representation of the consistent enrichment of YAP binding sites in ChIPSeq analysis of iPSC samples as compared to IgG control and input samples. ***Right:*** Piechart depicting the location annotation of YAP DNA binding sites as obtained by the ChIP-Seek web tool. Data are representative of four biological replicates. e) Barplot representation of the most significant Gene Ontology (GO) annotation categories obtained by ChIP-seq analysis of endogenous YAP binding targets in iPSCs: cell-matrix adhesion (GO:0007160), cell cycle (GO:0051726) and cell-cell adhesion (GO:0016337). f) Representative confocal images and relative quantification of panTEAD (red) staining at the center and edge of iPSCs micropatterned colonies. NANOG pluripotency marker is shown in green, nuclei are counterstained with DAPI (blue). g) Representation of the data obtained by analyzing the Elastic Modulus (or Young’s Modulus, *E*) of sparse or confluent hNDFs. *n*=10, ****P*<0.001.

**
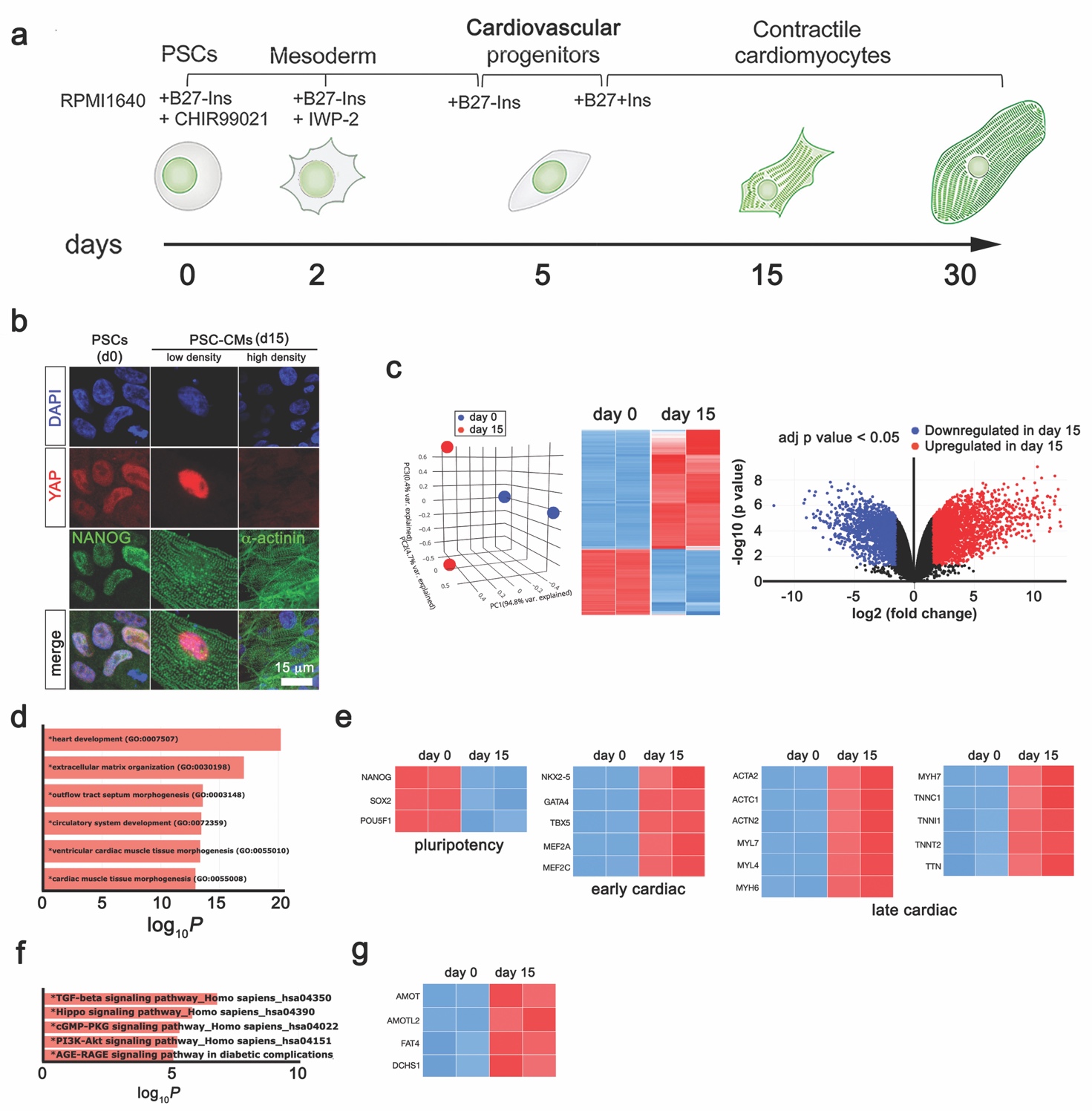
**

**Supplementary Figure 2. Genome-wide identification of YAP upstream regulators.** a) Graphical representation of the protocol used to obtain contractile cardiomyocytes from pluripotent stem cells. b) Representative confocal microscopy images of YAP expression (red) in human embryonic stem cells (PSCs) in the undifferentiated state (d0) or differentiated to contractile cardiomyocytes (PSC-CMs) for 15 days (d15) at low and high density. PSCs are stained for pluripotency marker NANOG (green) while PSC-CMs are decorated by cardiomyocyte-specific marker ALPHA SARCOMERIC ACTININ (α-actinin, green). Cell nuclei are counterstained by DAPI (blue). c) Principal Component Analysis (PCA, left), heatmap (center) and volcano plot (right) representation of the results obtained by comparing genome-wide RNA-sequencing of human embryonic stem cells (hESCs)-derived contractile cardiomyocytes (day 15) versus hESCs (day 0). d) Gene ontology (GO) annotation of the genes found differentially regulated between (hESCs)-derived contractile cardiomyocytes (day 15) and hESCs (day 0). e) Heatmap representation of genes significantly regulated in day 15 cells as compared to day 0 hESCs and belonging to the indicated categories. f) Gene ontology (GO) annotation of significantly regulated genes in day 15 cells versus day 0 hESCs belonging to the indicated pathways. g) Heatmap representation of genes differentially regulated in (hESCs)-derived contractile cardiomyocytes (day 15) versus day 0 hESCs identified as YAP upstream regulators. *n*=2.

**
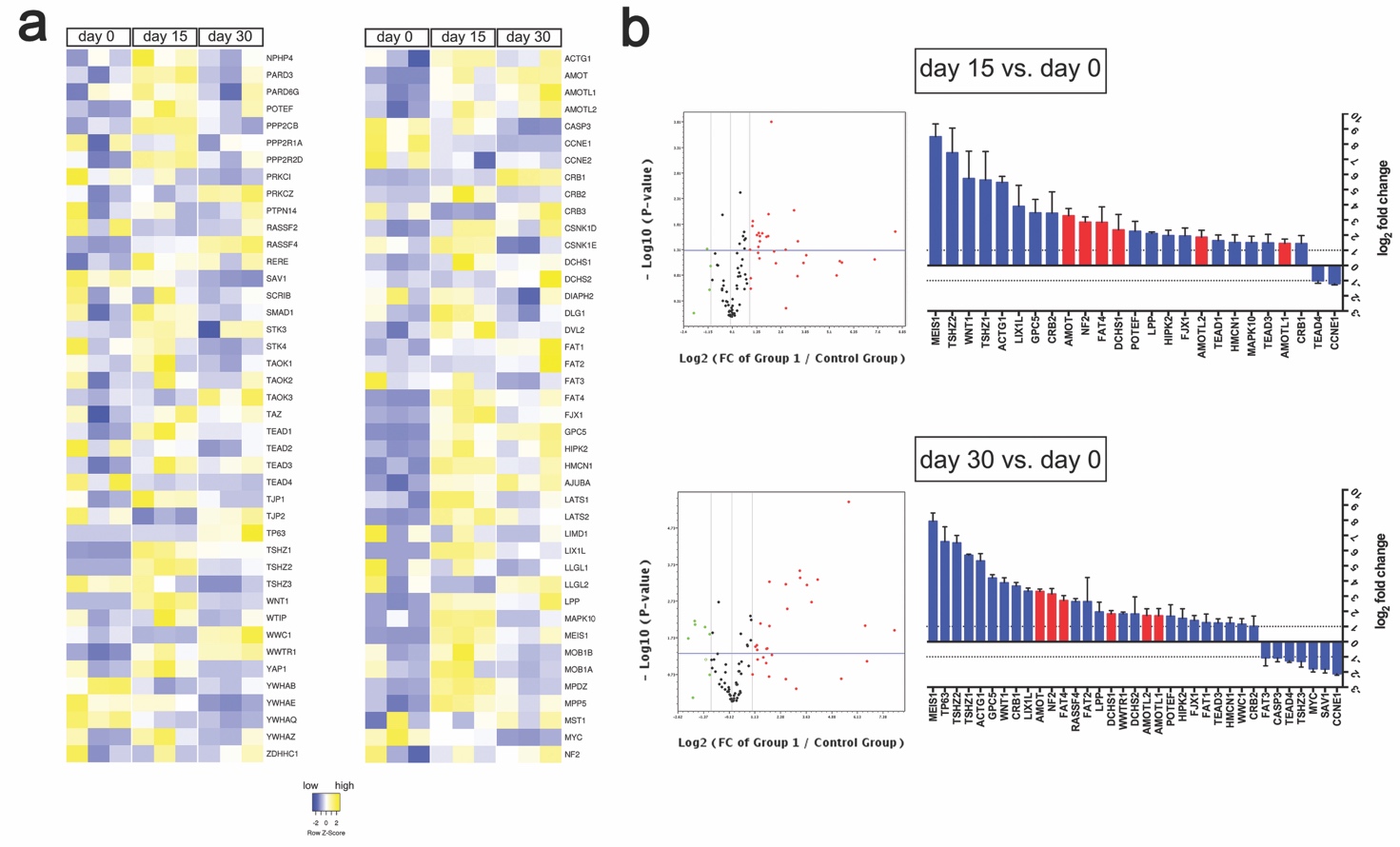
**

**Supplementary Figure 3. RT-qPCR identification of YAP upstream regulators**. a) Heatmap representation of Hippo pathway gene expression in undifferentiated iPSCs (day 0), day 15 and day 30 differentiated beating cardiomyocytes (iPSC-CMs), as obtained from RT-qPCR array (PAHS-172Z, QIAGEN). b) Volcano plot and barplot representation of significantly regulated genes of Hippo pathway in day 15 beating cardiomyocytes (top) and day 30 beating cardiomyocytes (bottom) as compared to undifferentiated iPSCs. *n*=3. *P* < 0.05. Log2Fc > |1|.

**
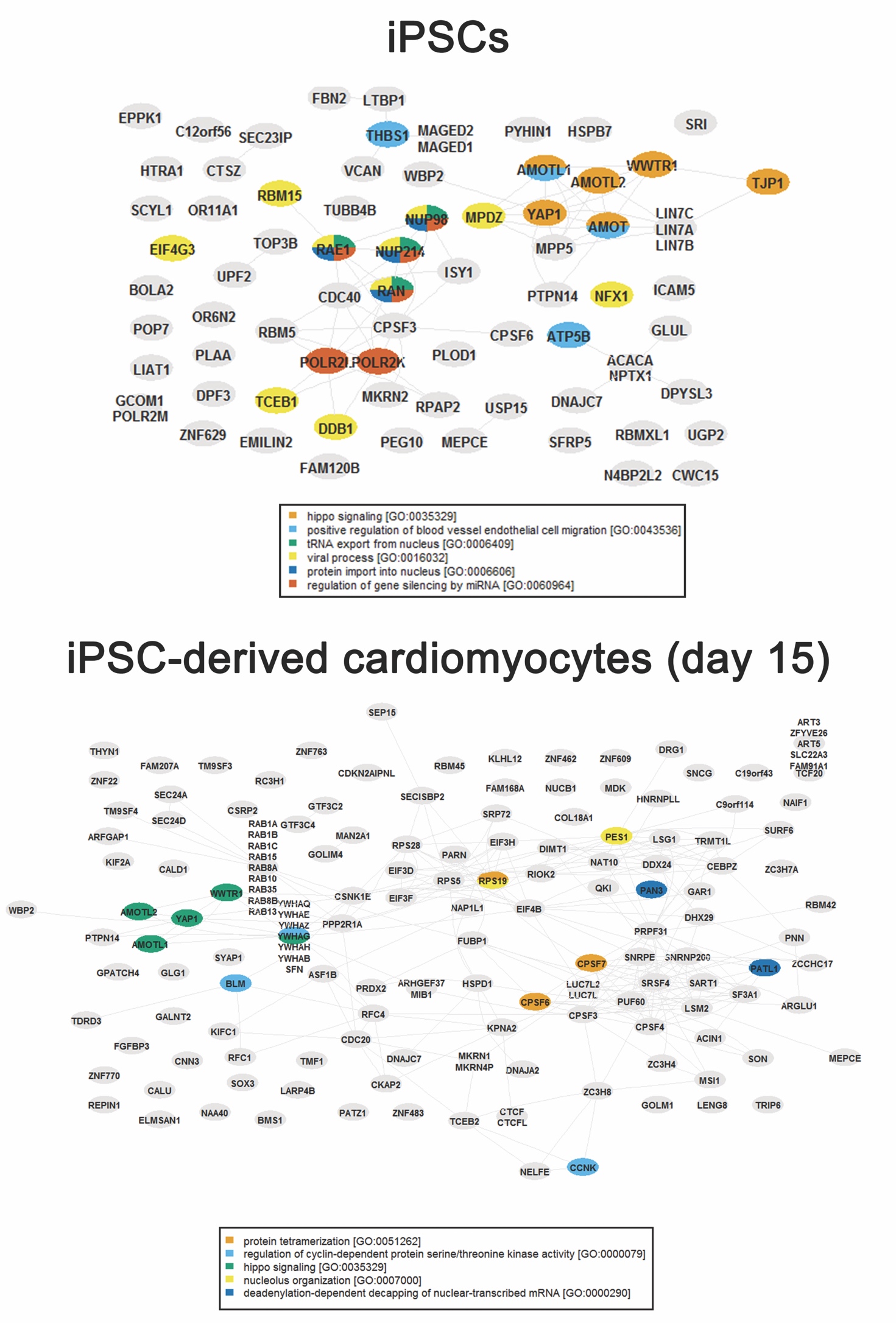
**

**Supplementary Figure 4. YAP interactome in iPSCs and iPSC-derived cardiomyocytes**. a) Medium-confidence protein-protein interactions as obtained from the STRING database (<https://string-db.org/>) in iPSCs (day 0). b) Medium-confidence protein-protein interactions as obtained from the STRING database (<https://string-db.org/>) in iPSC-CMs (day 15). Highlighted proteins belong to the pathways indicated below according to gene ontology (GO) annotation for biological process (*n*=7).

**
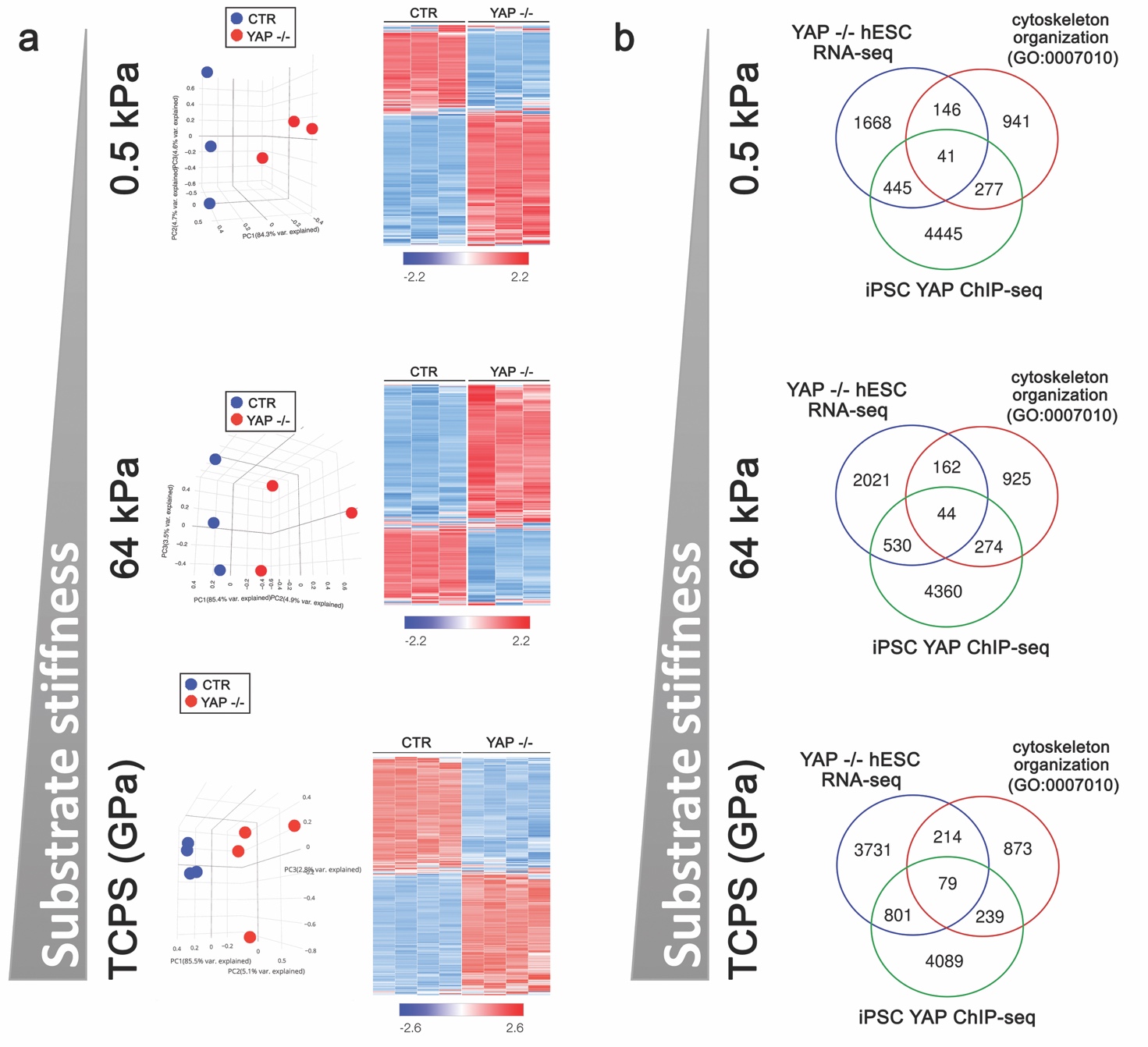
**

**Supplementary Figure 5.** Genome-wide analysis of YAP-dependent mechanosensitive genes. a) Principal Component Analysis (PCA, left) and heatmap (right) representation of the data obtained by differential RNA-seq analysis of CTR and YAP-/- hESCs grown onto substrates with increasing physiological stiffness (0,5 kPa, 64 kPa, n=3) and Tissue Culture Polystyrene (TCPS, *n*=4). Adjusted P < 0.05, logFc>|0.58|. b) Venn diagram representation of YAP *bona fide* targets being significantly regulated on substrates with controlled physiological stiffness and having an annotation for cytoskeleton organization (GO:0007010).

**
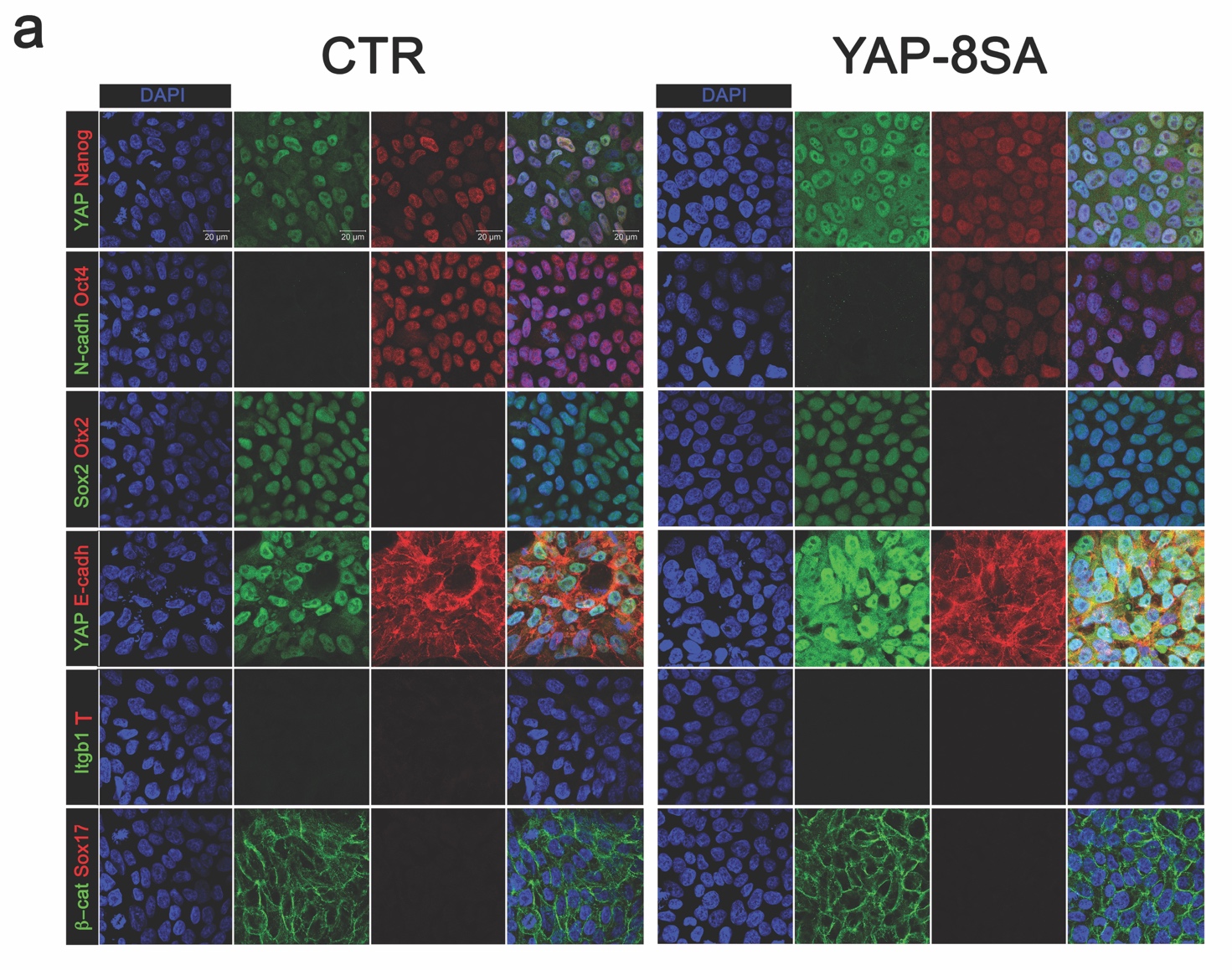
Supplementary Figure 6.** Generation of an iPSC line stably expressing YAP hyperactive form YAP-8SA. Extended confocal imaging characterization of YAP-8SA iPSCs as compared to isogenic iPSCs. Nuclei were counterstained with DAPI.

**
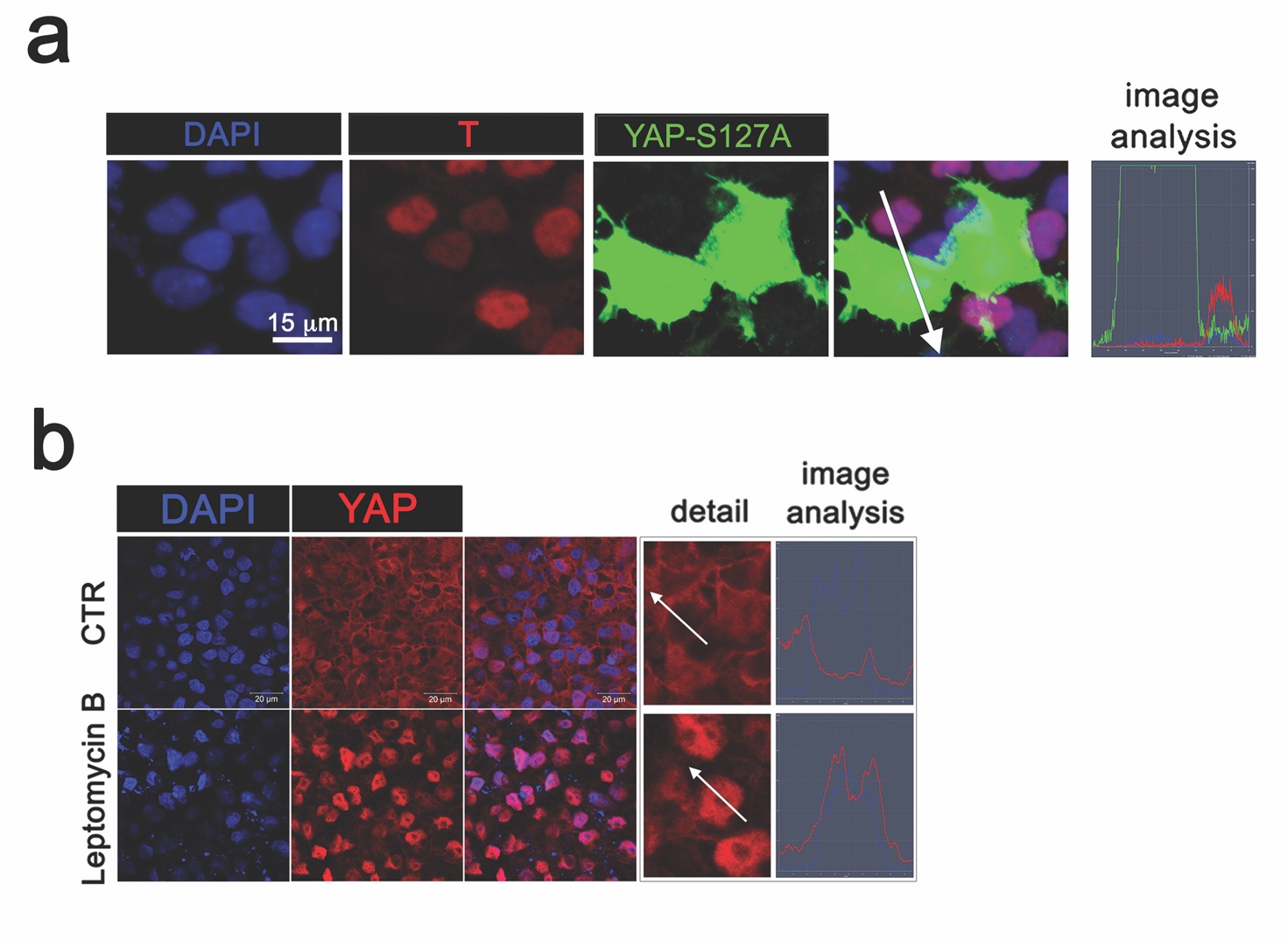
**

**Supplementary Figure 7.** YAP nuclear presence prevents mesoderm specification. a) Representative confocal images illustrating T (BRACHIURY) expression (red) in iPSCs co-transfected with YAP-S127A and GFP and induced to mesoderm specification for 2 days. The fluorescence intensity profile across the white arrows for red channel is shown in the graph. Nuclei were counterstained with DAPI. b) Representative confocal images illustrating YAP localization (red) in iPSCs treated or not with Leptomycin B (20 nm) and induced to mesoderm specification for 2 days. Nuclei were counterstained with DAPI.

**Supplementary Table 1.** List of genes identified as YAP binding targets in day 0 induced pluripotent stem cells (iPSCs) by ChIP-seq analysis.

**Supplementary Table 2:** Genes found significantly regulated in day 0 hESCs as compared to hESC-derived cardiomyocytes (day 15)

**Supplementary Table 3:** List of proteins identified as YAP interactors in day 0 iPSCs and day 15 iPSC-derived cardiomyocytes by Mass Spectrometry.

**Supplementary Table 4:** Genes found significantly regulated in CTR as compared to YAP -/- hESCs grown onto 0.5, 64 kPa or Tissue Culture PolyStyrene (TCPS) surfaces.

**Supplementary Table 5.** List of proteins belonging to the cytoskeleton pool found significantly regulated in CTR vs YAP -/ hESCs by Tandem Mass Tag Maas Spectrometry (TMT-MS).

**Supplementary Table 6.** List of primers used in the study.

**Supplementary Table 7.** List of antibodies used in the study.

**Supplementary video 1.** 3D reconstruction of F-actin (green) organization in CTR hESCs. Nuclei are counterstained with DAPI (blue).

**Supplementary video 2.** 3D reconstruction of F-actin (green) organization in YAP -/- hESCs. Nuclei are counterstained with DAPI (blue).

**Supplementary video 3.** 3D reconstruction of F-actin (green) organization in YAP -/- hESCs. Nuclei are counterstained with DAPI (blue).

**Supplementary References**

1. Palchesko RN, Zhang L, Sun Y, Feinberg AW. Development of Polydimethylsiloxane Substrates with Tunable Elastic Modulus to Study Cell Mechanobiology in Muscle and Nerve. PLoS ONE 2012; 7, e51499.
2. Rada V, Fila T, Zlámal P, Kytýř D, Koudelka P. Multi-channel control system for in-situ laboratory loading devices. Acta Polytech Proceed CTU Proceed 2018; 18**,** 15-19.
3. Caluori G, Pribyl J, Pesl M, Oliver-De La Cruz J, Nardone G, Skladal P, *et al*. Advanced and Rationalized Atomic Force Microscopy Analysis Unveils Specific Properties of Controlled Cell Mechanics. Front Physiol 2018; 9,1121.
4. Kim D, Pertea G, Trapnell C, Pimentel H, Kelley R, Salzberg SL. TopHat2: accurate alignment of transcriptomes in the presence of insertions, deletions and gene fusions. Genome Biol 2013; 14, R36.
5. Anders S, Pyl PT, Huber W. HTSeq—a Python framework to work with high-throughput sequencing data. Bioinformatics 2015; 31, 166-169.
6. Yu G, Wang L-G, Han Y, He Q-Y. clusterProfiler: an R Package for Comparing Biological Themes Among Gene Clusters. OMICS 2012; 16, 284-287.
7. Walter W, Sánchez-Cabo F, Ricote M. GOplot: an R package for visually combining expression data with functional analysis. Bioinformatics 2015; 31, 2912-2914.
8. Langmead B, Trapnell C, Pop M, Salzberg SL. Ultrafast and memory-efficient alignment of short DNA sequences to the human genome. Genome Biology 2009; 10, R25-R25.
9. Uusküla-Reimand L, Hou H, Samavarchi-Tehrani P, Rudan MV, Liang M, Medina-Rivera A, *et al*. Genome Biol**.** 2016; 17, 182-182.
10. Heinz S, Benner C, Spann N, Bertolino E, Lin YC, Laslo P, *et al*. Simple Combinations of Lineage-Determining Transcription Factors Prime cis-Regulatory Elements Required for Macrophage and B Cell Identities. Mol Cell 2010; 38, 576-589.
11. Chen T, Dent SYR. Chromatin modifiers and remodellers: regulators of cellular differentiation. Nat Rev Genet 2014; 15, 93-106.
12. Zambelli F, Pesole G, Pavesi G. PscanChIP: finding over-represented transcription factor-binding site motifs and their correlations in sequences from ChIP-Seq experiments. Nucleic Acids Research 2013; 41, W535- W543.
13. Stejskal K, Potěšil D, Zdráhal Z. Suppression of Peptide sample losses in autosampler vials. Journal of proteome research 2013; 12, 3057-3062.
14. Silva JC, Gorenstein MV, Li GZ, Vissers JP, Geromanos SJ. Absolute Quantification of Proteins by LCMS E. Molecular & Cellular Proteomics 2006; 5, 144-156.
15. Wiśniewski JR, Zougman A, Nagaraj N, Mann M. Universal sample preparation method for proteome analysis. Nat Methods 2009; 6, 359-362.
16. Leavesley SJ, Britain AL, Cichon LK, Nikolaev VO, Rich TC. Assessing FRET using spectral techniques. Cytometry A 2013; 83, 898-912.
17. Plotnikov SV, Sabass B, Schwarz US, Waterman CM. High-Resolution Traction Force Microscopy. Methods Cell Biol 2014; 123, 367-394.
18. Han SJ, Oak Y, Groisman A, Danuser G. Traction microscopy to identify force modulation in subresolution adhesions. Nature Methods 2015; 12, 653-656.
19. Schneider CA, Rasband WS, Eliceiri KW. NIH Image to ImageJ: 25 years of image analysis. Nat Methods 2012; 9, 671-675.
20. Legland D, Arganda-Carreras I, Andrey P. MorphoLibJ: integrated library and plugins for mathematical morphology with ImageJ. Bioinformatics 2016; 32, 3532-3534.
21. Conway JR, Lex A, Gehlenborg N. UpSetR: an R package for the visualization of intersecting sets and their properties. Bioinformatics 2017; 33, 2938-2940.
