# Supplementary material for: YAP–TEAD1 control of cytoskeleton dynamics and intracellular tension guides human pluripotent stem cell mesoderm specification
Source: Cell Death Differ. 2020 Oct 28;28(4):1193–207. doi: 10.1038/s41418-020-00643-5 (PMC8027678; doi:10.1038/s41418-020-00643-5)
Supplement: Supplementary file 7 — Supplementary Table 6 [file 41418_2020_643_MOESM7_ESM.docx]

| Gene | Forward 5’-3’ | Reverse 5’-3’ |
| --- | --- | --- |
| AMOT | TCCAATGCTGGATCAGGCTTGC | ATTCAGCACGGTTAGTCTCCACC |
| NANOG | GGTCTCGTATTTGCTGCATC | ACTCGGTGAAATCAGGGTAA |
| OCT4 | GGCAACCTGGAGAATTTGTT | TTACAGAACCACACTCGGAC |
| SOX2 | CATCACCCACAGCAAATGAC | GAAGTCCAGGATCTCTCTCA |
| MESP1 | CTCTGTTGGAGACCTGGATG | CCTGCTTGCCTCAAAGTG |
| T | ATCACCAGCCACTGCTTC | GGGTTCCTCCATCATCTCTT |
| EOMES | AACTCCATCTCCCACGGATT | AAGTGTTGACAAAGGGCTCC |
| WNT 3 | AGCTGCCAGGAGTGTAT | TCCTGCTTCCCATGAGA |
| GAPDH | AAGTATGACAACAGCCTCAA | TCCTTCCACGATACCAAAGT |

**Supplementary Table 6.** List of primers used in the study.
